# Defibrillation effectiveness and safety of the shock waveform used in a contemporary wearable cardioverter defibrillator: Results from animal and human studies

**DOI:** 10.1371/journal.pone.0281340

**Published:** 2023-03-14

**Authors:** Marye J. Gleva, Joseph Sullivan, Thomas C. Crawford, Greg Walcott, Ulrika Birgersdotter-Green, Kelley R. Branch, Rahul N. Doshi, Kaisa Kivilaid, Kelly Brennan, Ron K. Rowbotham, Laura M. Gustavson, Jeanne E. Poole

**Affiliations:** 1 Division of Cardiology, Department of Medicine, Washington University in Saint Louis School of Medicine in Saint Louis, St. Louis, Missouri, United States of America; 2 Kestra Medical Technologies, Inc., Redmond, Washington, United States of America; 3 Division of Cardiology, Department of Medicine, University of Michigan, Ann Arbor, Michigan, United States of America; 4 Division of Cardiovascular Diseases, Department of Medicine, University of Alabama at Birmingham, Birmingham, Alabama, United States of America; 5 University of California San Diego, San Diego, CA, United States of America; 6 Division of Cardiology, Department of Medicine, University of Washington School of Medicine, Seattle, Washington, United States of America; 7 Honor Health, Scottsdale, AZ, United States of America; 8 Labcorp Inc., Minneapolis, MN, United States of America; University of Messina, ITALY

## Abstract

**Introduction:**

The wearable cardioverter defibrillator (WCD) is used to protect patients at risk for sudden cardiac arrest. We examined defibrillation efficacy and safety of a biphasic truncated exponential waveform designed for use in a contemporary WCD in three animal studies and a human study.

**Methods:**

Animal (swine) studies:

#1: Efficacy comparison of a 170J BTE waveform (SHOCK A) to a 150J BTE waveform (SHOCK B) that approximates another commercially available waveform. Primary endpoint first shock success rate.

#2: Efficacy comparison of the two waveforms at attenuated charge voltages in swine at three prespecified impedances. Primary endpoint first shock success rate.

#3: Safety comparison of SHOCK A and SHOCK B in swine. Primary endpoint cardiac biomarker level changes baseline to 6 and 24 hours post-shock.

Human Study: Efficacy comparison of SHOCK A to prespecified goal and safety evaluation. Primary endpoint cumulative first and second shock success rate. Safety endpoint adverse events.

**Results:**

Animal Studies

#1: 120 VF episodes in six swine. First shock success rates for SHOCK A and SHOCK B were 100%; SHOCK A non-inferior to SHOCK B (entire 95% CI of rate difference above -10% margin, p < .001).

#2: 2,160 VF episodes in thirty-six swine. Attenuated SHOCK A was non-inferior to attenuated SHOCK B at each impedance (entire 95% CI of rate difference above -10% margin, p < .001).

#3: Ten swine, five shocked five times each with SHOCK A, five shocked five times each with SHOCK B. No significant difference in troponin I (p = 0.658) or creatine phosphokinase (p = 0.855) changes from baseline between SHOCK A and SHOCK B.

Human Study: Thirteen patients, 100% VF conversion rate. Mild skin irritation from adhesive defibrillation pads in three patients.

**Conclusions:**

The BTE waveform effectively and safely terminated induced VF in swine and a small sample in humans.

**Trial registration:**

**Human study clinical trial registration:** URL: https://clinicaltrials.gov; Unique identifier: NCT04132466.

## Introduction

A wearable cardioverter defibrillator (WCD) is an external defibrillator capable of automatic detection and treatment of life-threatening ventricular tachyarrhythmias. Contemporary external defibrillators utilize biphasic truncated exponential (BTE) impedance compensated waveforms that deliver between 150J and 360J [1, 2]. The efficacy of BTE waveforms from multiple external devices, including a WCD, has been thoroughly established in clinical trials [3–14]. Until recently only one WCD delivering 150J was approved for commercial use in the United States [15]. Another WCD, the ASSURE WCD System (Kestra Medical Technologies, Inc., Kirkland WA), has recently been developed that delivers a 170J BTE waveform utilizing a 140 μF capacitor [16].

Historically, shock success rates for induced ventricular fibrillation (VF) have been used to assess defibrillation efficacy in both animals and humans [6, 17]. As the commercially available devices have different waveform parameters and different nominal energies, a demonstration of defibrillation efficacy is clinically relevant to prescribing physicians. Additionally, potential cardiac injury after defibrillation shock has been a recognized safety concern; Troponin I and creatine phosphokinase (CPK-MB) serum biomarker levels provide one way of assessing cardiac injury. We designed three animal studies and one human study to evaluate the new BTE waveform. Our first objective was to evaluate defibrillation efficacy and safety in animals. Two comparative animal studies were conducted to quantify shock success rates. A third comparative animal study measured changes in troponin I and CPK-MB levels following defibrillation shocks. Our second objective was to demonstrate shock success rate and safety in humans. Results of these four studies provide the evidence for defibrillation safety and efficacy while minimizing risk to humans.

## Methods

**Fig 1:**
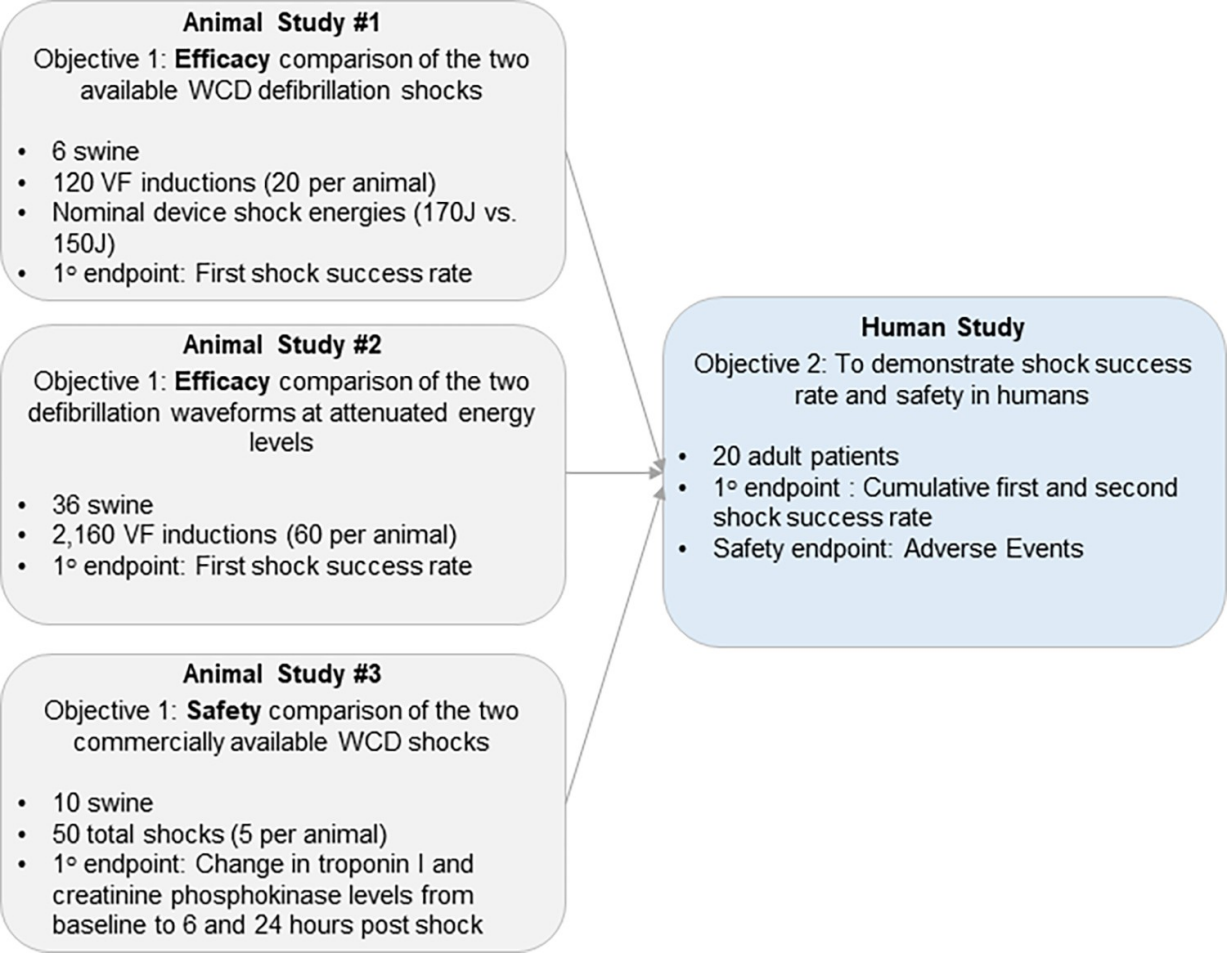
Flowchart of the defibrillation safety and efficacy studies. Flowchart describing objectives, endpoints and sample sizes for the three animal studies and one human study providing a comprehensive assessment of defibrillation safety and efficacy of the new BTE waveform designed for use in a WCD.

The objectives and endpoints for the four studies are illustrated in Fig 1.

For the animal studies, we compared the new BTE waveform with the BTE waveform that approximates that of another commercially available BTE waveform. A detailed description of both waveforms is provided below. Subsequently, the new BTE waveform was evaluated in a human study.

### Waveform descriptions

**Fig 2:**
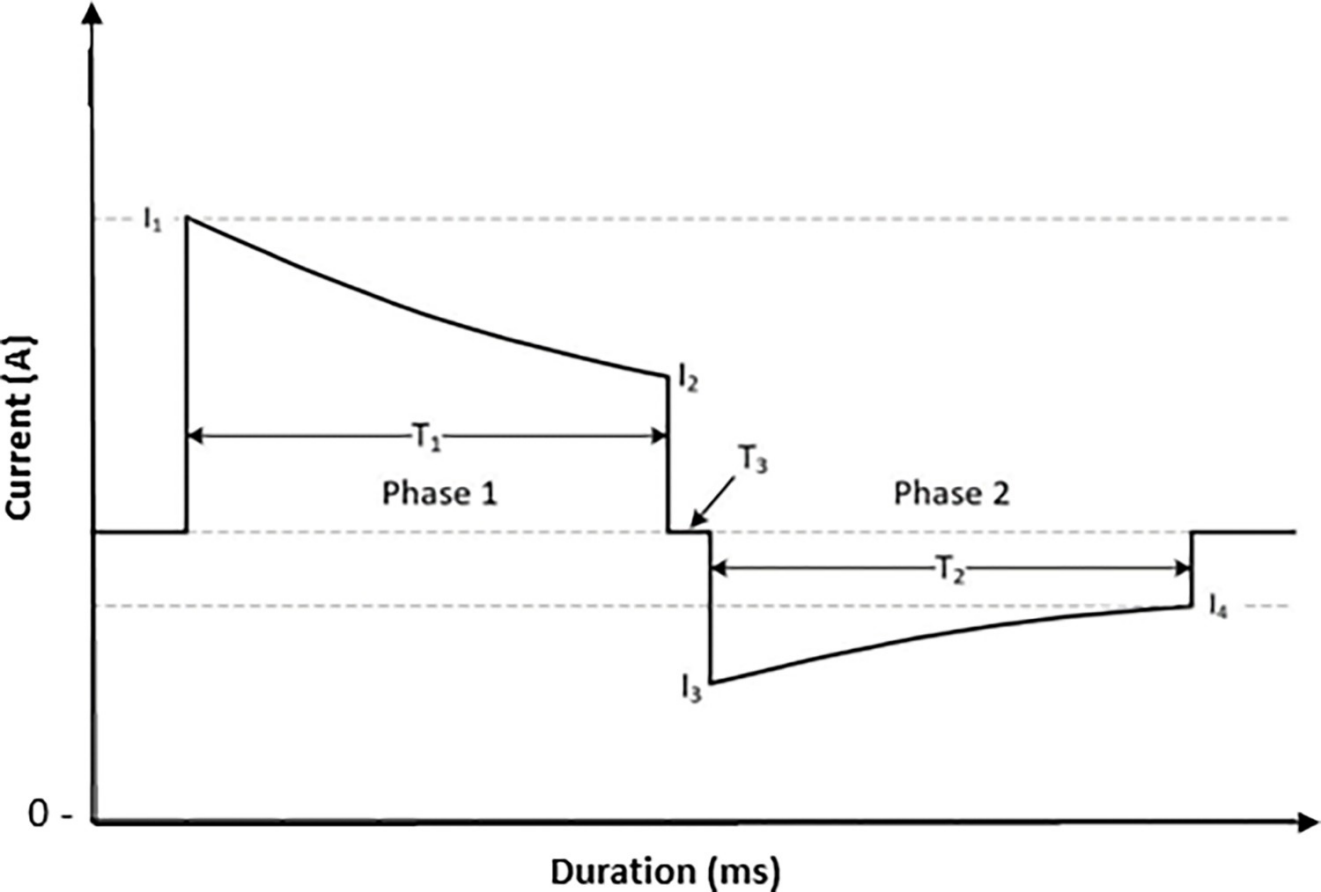
Typical Biphasic Truncated Exponential (BTE) shock waveform. Graphical **r**epresentation of the typical BTE waveform showing current as a function of time. I1 = Peak current, I2 = Phase 1 trailing edge current, I3 = Phase 2 peak current, I4 = Phase 2 trailing edge current, T1 = Phase 1 period, T2 = Phase 2 period, T3 = Interphase delay.

The new BTE waveform, hereafter referred to as SHOCK A, was designed to deliver 170J into 50 ohms utilizing a 140 μF capacitor. Fifty (50) ohms was used because it is the industry standard for defibrillation testing [18]. Hardware design limited the maximum charge voltage to approximately 1700V. These voltage and energy requirements dictated the appropriate capacitor value. Phase durations of a typical BTE waveform are shown in Fig 2. SHOCK A phase durations were designed to increase with increasing impedance and are truncated at less than 80% tilt to be consistent with other commercially available BTE waveforms [19]. Phase 2 was set equal to phase 1 at low impedances and shorter than phase 1 at high impedances, an approach that has been validated for at least one external defibrillator [20]. The impedance is calculated during the initial phase of the shock based on the rate of decay of the capacitor voltage. Given these constraints, SHOCK A has a nominal operating charge voltage of 1660V to deliver 170J into 50 ohms. The charge voltage divided by patient resistance determines the peak current (I_1_).

The comparator BTE waveform, hereafter referred to as SHOCK B, was generated with a nominal charge voltage of 1550V to deliver approximately 150J into 50 ohms, assuming a 175 μF capacitor. In SHOCK B, the phase 1 duration increases with increasing impedance and the phase 2 duration is fixed, delivering an overall tilt less than 60%. The specific SHOCK A and SHOCK B waveform parameters are shown in Table 1. Two custom waveform generators were built using these specifications to deliver these waveforms for the animal studies. The waveform generators include a capacitor, capacitor charger, and switching circuitry to deliver the respective biphasic waveforms.

**Table 1:** SHOCK A and SHOCK B waveform parameters.

Load Impedance	Charge Voltage	Peak Current (I_1_)	Phase 1 (T_1_)	Phase 2 (T_2_)	Tilt (I_1_-I_4_)/I_1_	Delivered Energy
**SHOCK A Waveform Parameters (140 μF capacitor)**						
50 Ω	1660V	32 A	5.0 ms	5.0 ms	76%	170 J
85 Ω	1660V	19 A	7.2 ms	7.2 ms	70%	165 J
125 Ω	1660V	13 A	8.8 ms	5.3 ms	56%	146 J
**SHOCK B Waveform Parameters (175 μF capacitor)**						
50 Ω	1550V	30 A	4.1 ms	2.5 ms	55%	145 J
85 Ω	1550V	17 A	8.1 ms	2.5 ms	53%	141 J
125 Ω	1550V	12 A	12.7 ms	2.5 ms	52%	140 J

Ω = ohms; A = amps; ms = milliseconds; J = Joules; V = Volts

### Animal studies

Three animal studies were performed to evaluate the defibrillation efficacy and safety of SHOCK A and SHOCK B. These studies were conducted in accordance with the National Research Council (US) Institute for Laboratory Animal Research Guide for the Care and Use of Laboratory Animals [21]. All three animal study protocols were reviewed and approved by the University of Alabama Institutional Animal Care and Use Committee (UAB IACUC Animal Project Number 20940, June 2017). *Sus Scrofa Domesticus* swine were selected as the animal model because their thoracic anatomy, coronary arteries, and thoracic impedance are similar to humans and they have been used in other defibrillation studies [17, 22]. Non-human primates were not used in any of the animal studies. All efforts were made to treat animals humanely and to minimize suffering. Animals were euthanized under anesthesia by inducing ventricular fibrillation. Cardiac standstill was confirmed with bilateral thoracotomies and manual palpation.

#### Animal Study #1

**Fig 3:**
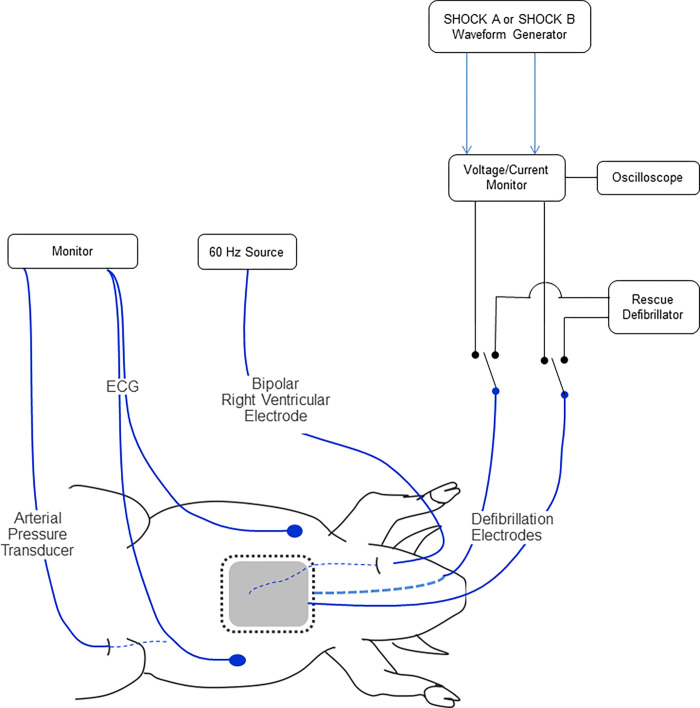
Animal Study #1 experimental setup. Image illustrating laboratory setup to enable ventricular fibrillation (VF) induction, monitoring and defibrillation in swine. ECG = Electrocardiogram.

Animal Study #1 (protocol number 3338359) compared success rates of SHOCK A (170J) and SHOCK B (150J). The energies of these two shocks were chosen specifically because they reflect the nominal energies available in commercially available WCDs. The experimental setup is illustrated in Fig 3.

Swine (~30 kg) were premedicated with intramuscular injections of telazol (~4.4 mg/kg) and xylazine (~4.4 mg/kg). Animals were placed on a surgical table and then intubated with a cuffed endotracheal tube and anesthetized with isofluorane (1–5% inhalation). The lateral sides of the thorax were shaved for placement of defibrillation electrodes (QUICK-COMBO^TM^ Model 11996; Physio-Control, Redmond, WA) in the lateral-lateral positions. Each pad has an active area of 115 cm^2^. A vein in the ear was isolated and a catheter was placed for saline administration to keep the animals hydrated. An ECG was recorded using commercially available equipment. Animals were maintained at a temperature of 37 ± 3°C throughout the experiment. An oscilloscope with a voltage and current monitor was used to record each shock. VF was electrically induced using 60Hz current and an investigational defibrillation shock was delivered after 10 seconds of sustained VF. A rescue defibrillator was available to convert the rhythm in the case SHOCK A or SHOCK B failed.

**Fig 4:**
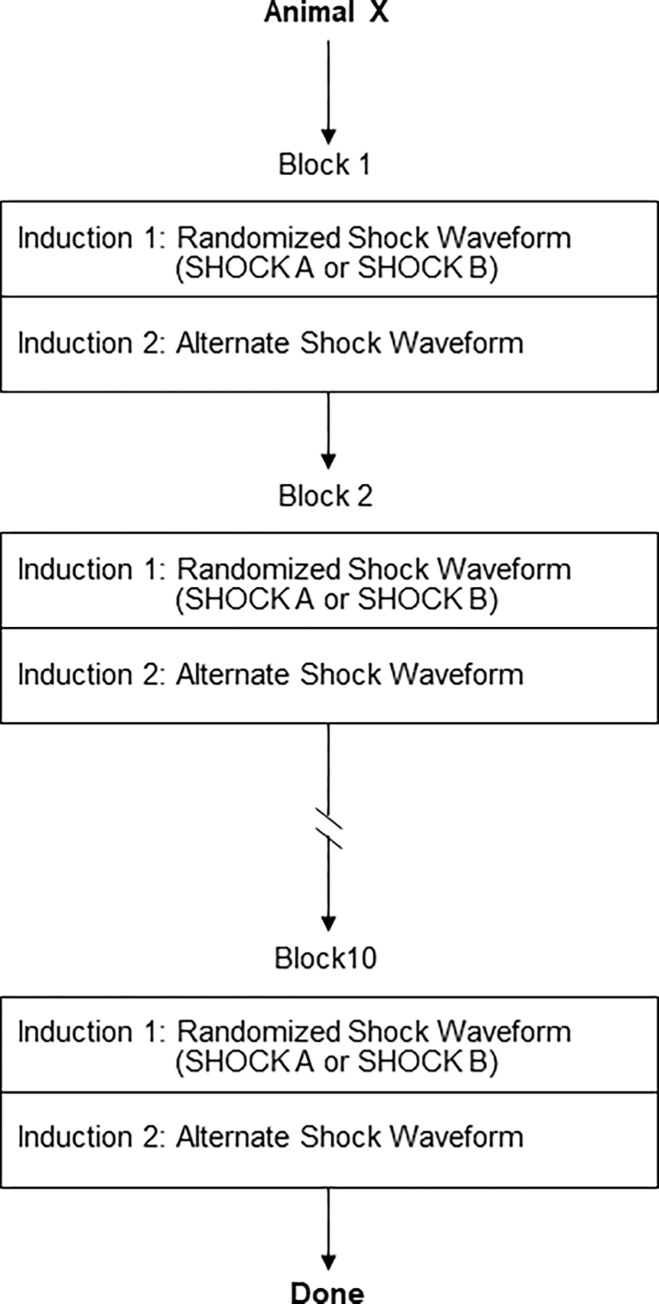
Animal Study #1 shock sequence. Figure illustrating shock sequence and randomization of shock type for each animal.

A total of 20 VF inductions were performed in each animal. The VF inductions were performed in blocks. Each block included two inductions. The sequence of shock delivery within each block, SHOCK A (170J) or SHOCK B (150J), was randomized (Fig 4). Ten blocks were performed in each animal.

Statistical analysis for Animal Study #1: The primary endpoint was a non-inferiority comparison of the first shock success rates. Shock success was defined as termination of VF. Shock A was considered non-inferior to Shock B if the entire 95% confidence interval for the success rate difference (SHOCK A–SHOCK B) was above the -10% non-inferiority margin. The -10% margin was based on prior published international standards for defibrillation shock success [23]. The shock success rates for SHOCK A and SHOCK B respectively were calculated as the number of successful shocks divided by the total number shocks delivered. A sample of six animals provided an estimated power of more than 95%.

#### Animal Study #2

Animal Study #2 (protocol number 3337920) was undertaken to address the possibility that Animal Study #1 might not detect a potential difference in shock efficacy. We hypothesized that if the charge voltages (and resulting energies) of both waveforms were attenuated (reduced) proportionally, a difference in first shock success rates might be observed. Recognizing the differences between SHOCK A and SHOCK B parameters as shown in Table 1, we held the ratio between the charge voltages constant. The nominal charge voltage for SHOCK B is 1550V and for SHOCK A is 1660V providing a charge voltage ratio of 0.93. The resulting test shocks in Animal Study #2 used attenuated charge voltages designated as attSHOCK A and attSHOCK B.

This study was performed at three prespecified impedances of 50, 85 and 125 ohms to encompass the range of shock impedances observed in out-of-hospital cardiac arrest patients [24]. The charge voltages for attSHOCK A and attSHOCK B were determined separately for each animal at each impedance. We hypothesized that this novel approach of attenuated waveforms tested at multiple prespecified impedances would be more likely to demonstrate measurable differences in defibrillation efficacy.

**Fig 5:**
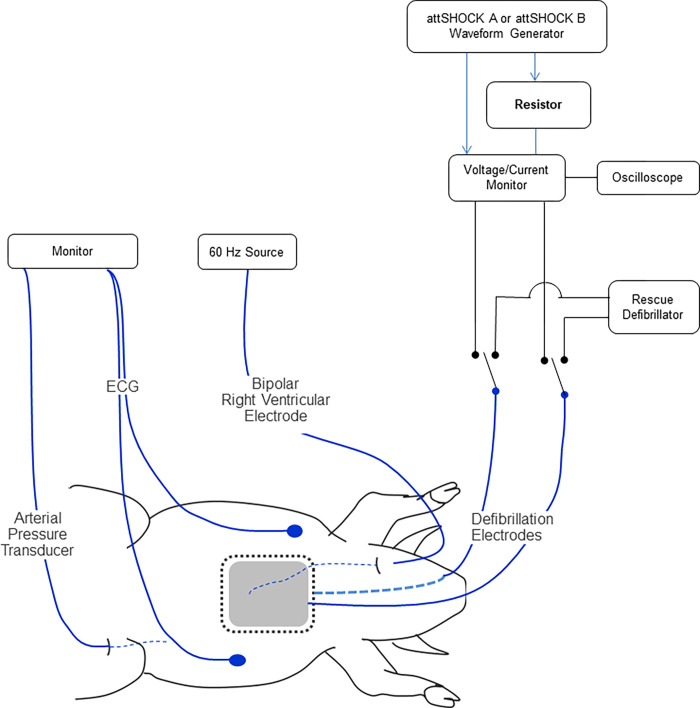
Animal Study #2 experimental setup. Image illustrating laboratory setup to enable ventricular fibrillation (VF) induction, monitoring and defibrillation in swine. ECG = Electrocardiogram, att = attenuated.

The experimental setup for Animal Study #2 is illustrated in Fig 5. Resistors were added in series with each animal to achieve a combined impedance that matched each of the three prespecified impedances (50, 85 and 125 ohms). The same resistors were used for attSHOCK A and attSHOCK B for each animal at each impedance level, so the fraction of the energy delivered to each animal was the same.

Swine were premedicated with intramuscular injections of telazol (~4.4 mg/kg) and xylazine (~4.4 mg/kg). Small swine (~10 kg) were selected to ensure the delivered energy was sufficient for termination of VF at each impedance. Animals were placed on a surgical table and then intubated with a cuffed endotracheal tube and anesthetized with isofluorane (1–5% inhalation). The thorax was shaved to allow for anterior-posterior placement of defibrillation electrodes (Pediatric QUICK-COMBO^TM^ Model 11996–000093; Physio-Control, Redmond, WA; active area 52cm^2^). Anterior-posterior placement was chosen due to the small size of the animals. A vein in the ear was isolated and a catheter was placed for saline administration to keep the animals hydrated. An ECG was recorded using commercially available equipment. Animals were maintained at a temperature of 37 ± 3°C throughout the experiment. An oscilloscope with a voltage and current monitor was used to record each shock. VF was electrically induced using 60Hz current and an investigational defibrillation shock was delivered after 10 seconds of sustained VF. A rescue defibrillator was available to convert the rhythm in the case attSHOCK A or attSHOCK B failed.

Dixon’s up-and-down small sample method [25] as applied by McDaniel et al. [24] was used to establish the attSHOCK A charge voltage where 50% defibrillation success could be achieved. The charge voltage was determined for each animal at each of the three target impedances. Then, for each resulting attSHOCK A charge voltage, the corresponding attSHOCK B charge voltage was calculated by multiplying by 0.93. Once these six charge voltages (attSHOCK A and attSHOCK B at each of three target impedances) had been established for the animal, we then proceeded to the randomized test shock sequence.

**Fig 6:**
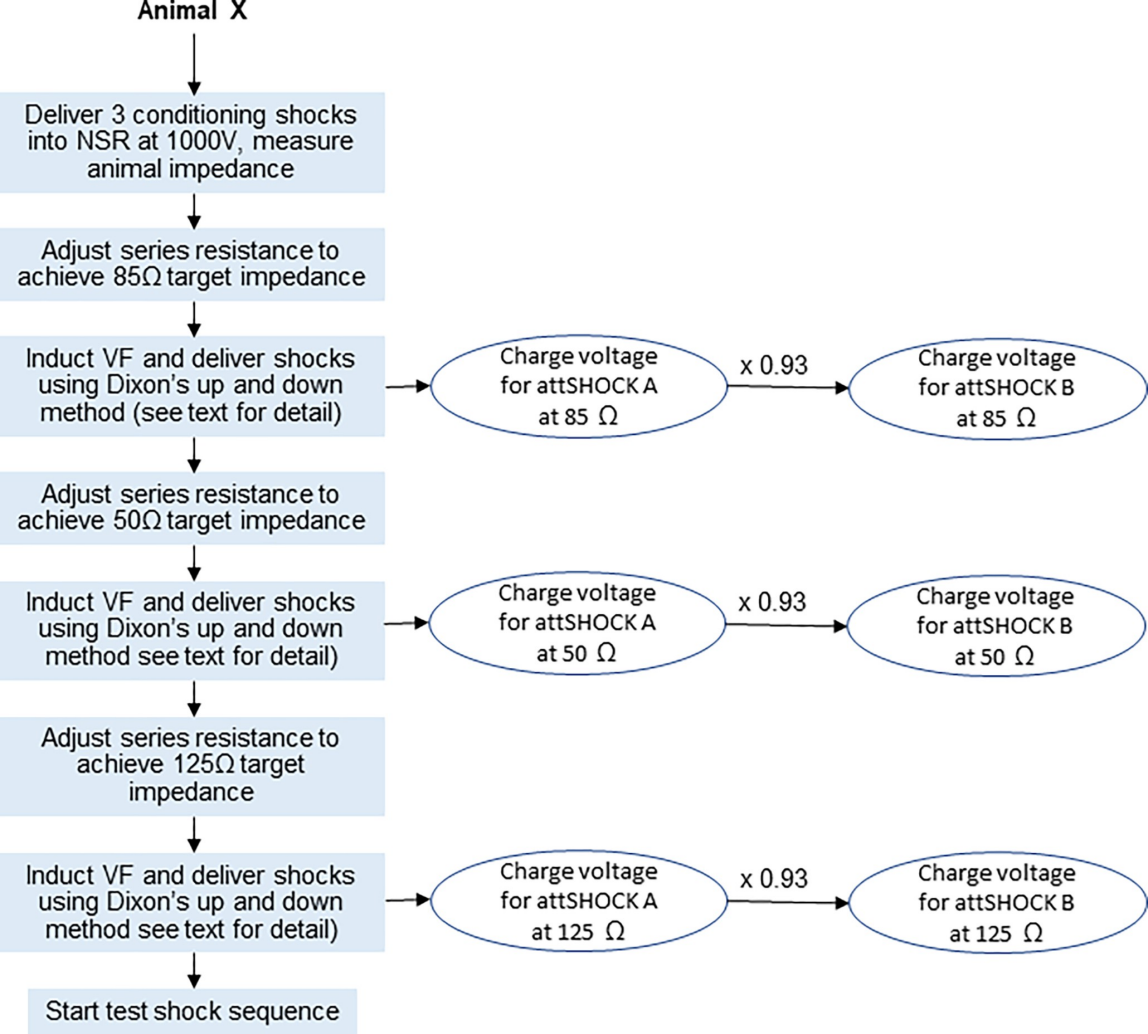
Animal Study #2 charge voltage determination. Image illustrating the process to establish the charge voltage for 50% defibrillation success of attSHOCK A and the subsequent calculation of the charge voltage for attSHOCK B at each target impedance. NSR = normal sinus rhythm, att = attenuated.

Prior to determining the charge voltages for attSHOCK A and attSHOCK B, three 1,000V conditioning shocks were delivered during normal sinus rhythm to stabilize and measure the initial impedance of the animal (Fig 6). Dixon’s up and down method was then performed as follows: The charge voltage for the first attSHOCK A was set as near as possible to the estimated dose at which 50% success was expected. VF was then induced, and the initial attSHOCK A was delivered. If the shock was successful, the charge voltage of the subsequent attSHOCK A was decreased. If the shock was unsuccessful, a rescue shock was delivered and the charge voltage of the subsequent attSHOCK A was increased for the next induction. Charge voltages were increased/decreased in 5% steps on a log voltage scale. A change in shock success constituted a “reversal”. The induction and shock sequence was repeated until three shocks had been delivered following the first reversal. The step following the third shock established the charge voltage for attSHOCK A at that impedance. The resulting attenuated charge voltage provided approximately 50% defibrillation shock success rate. The charge voltage for attSHOCK B at that impedance was then determined by multiplying the attSHOCK A charge voltage by 0.93.

**Fig 7:**
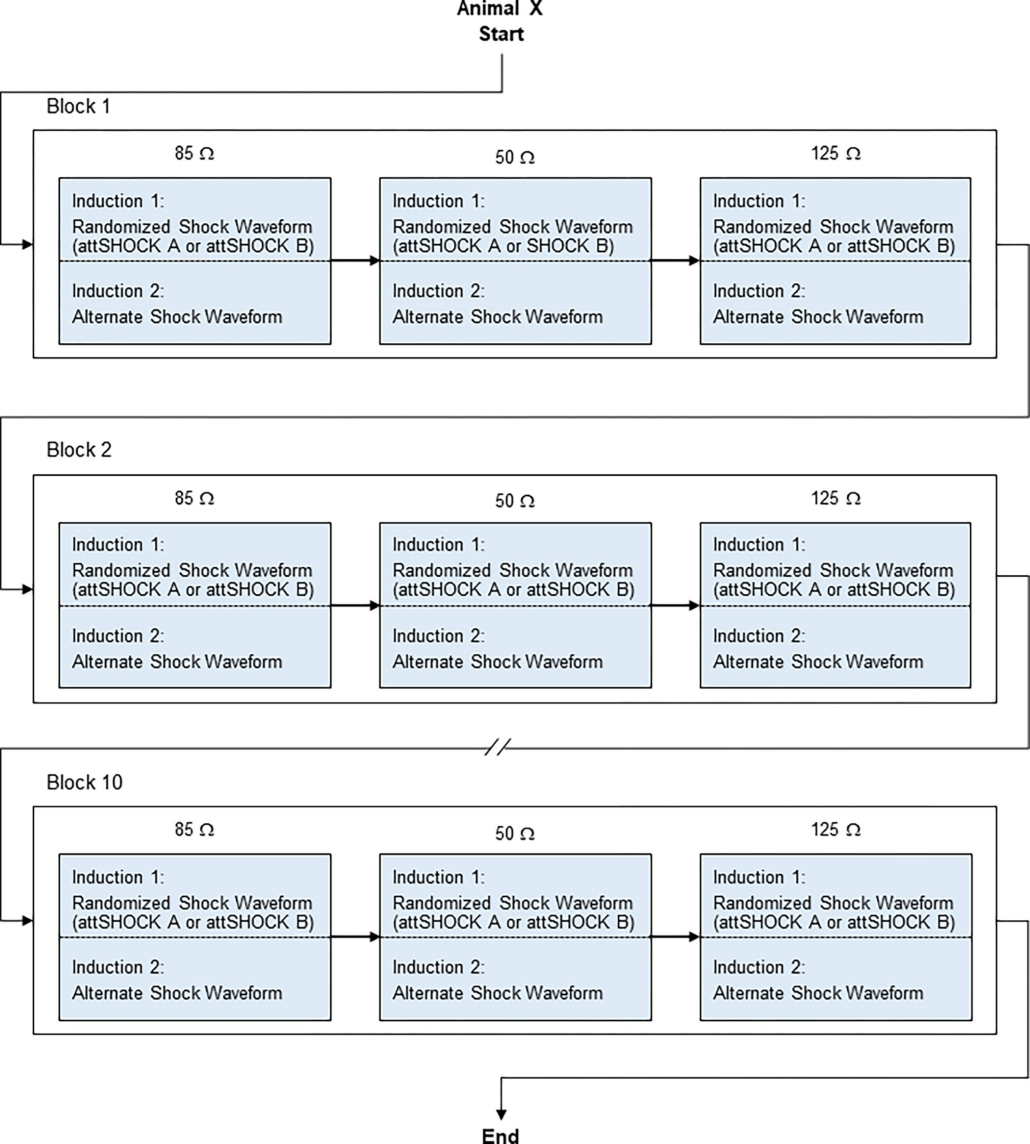
Animal Study #2 shock sequence. Figure illustrating attSHOCK A and attSHOCK B defibrillation shock sequence for each animal. att = attenuated.

The randomized defibrillation test shock sequence was then initiated, which included a total of 60 VF inductions performed in each animal (Fig 7). The VF inductions were performed in blocks of 6. Ten blocks were performed in each animal. The order of test shock delivery, attSHOCK A or attSHOCK B, within each block at each impedance was randomized.

Statistical analysis for Animal Study #2: The primary endpoint was a non-inferiority comparison of the first shock success rates of attSHOCK A and attSHOCK B. Shock success was defined as termination of VF. Non-inferiority was demonstrated if the entire 95% confidence interval of the difference in first shock success rates (attSHOCK A–attSHOCK B) was above the -10% non-inferiority margin. The shock success rates for attSHOCK A and attSHOCK B respectively were calculated as the number of successful shocks divided by the total number shocks delivered. The Wilcoxon signed-rank test was to be used to determine the lower bound of the 95% confidence interval of the difference in shock conversion success rates. A sample size of 36 animals was determined to provide 80% power to reject the null hypothesis for non-inferiority. If non-inferiority was demonstrated, then superiority at each impedance value was assessed in *post hoc* analysis using the same confidence interval method but comparing the lower bound of the confidence interval to 0%.

#### Animal Study #3

**Fig 8:**
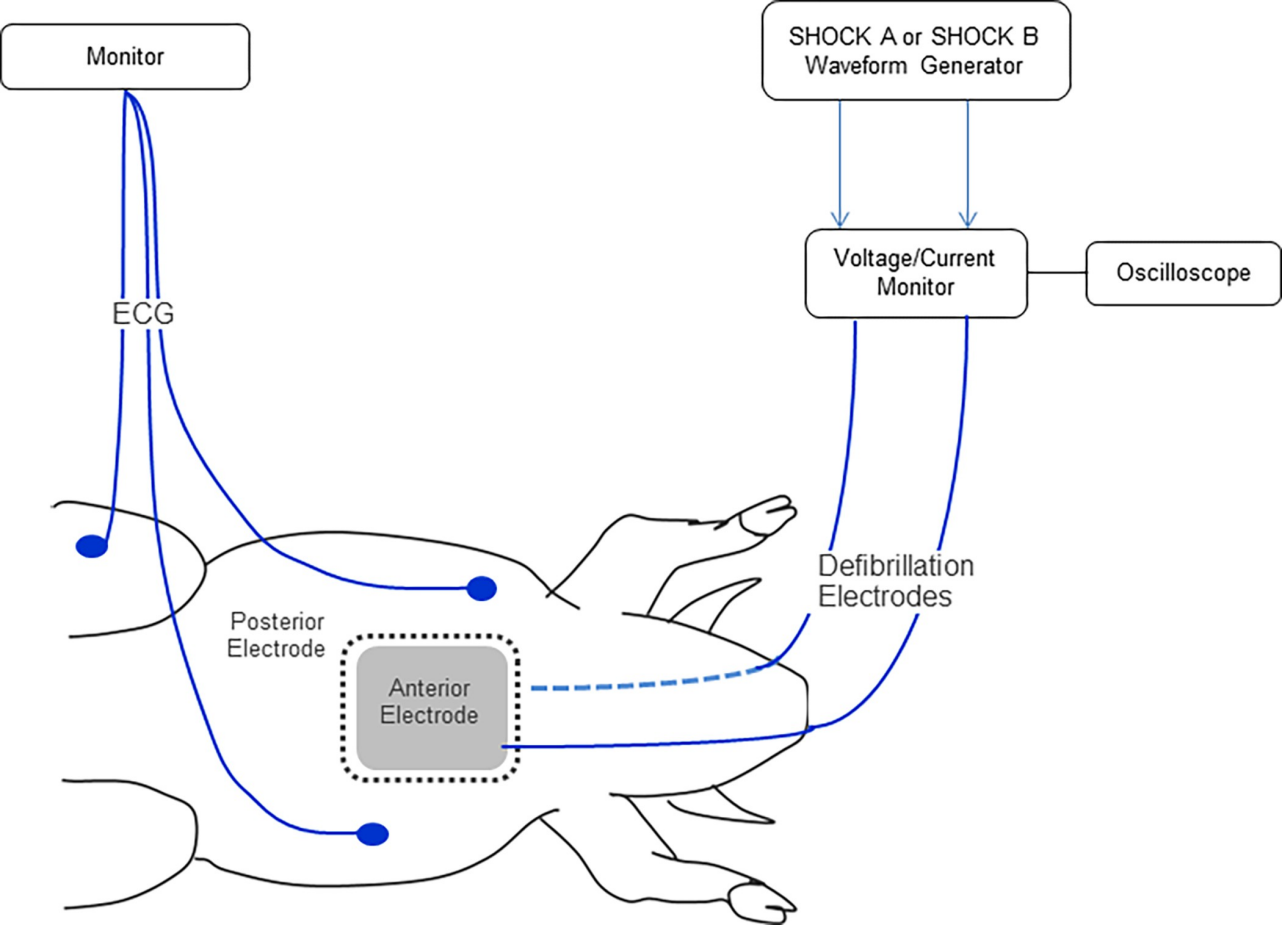
Animal Study #3 experimental setup. Image illustrating laboratory setup to enable shock delivery during normal sinus rhythm and monitoring in swine. ECG = Electrocardiogram.

While Animal Studies #1 and #2 compared shock success rates, Animal Study #3 (protocol number DHF-00046-01) evaluated potential cardiac muscle injury by measuring biomarker levels before and after shocks with SHOCK A or SHOCK B. Biomarkers included troponin I (Ohio State University Reference Lab, Siemens) and creatine phosphokinase iso-enzymes (CPK-MB Antech Diagnostics, Beckman Coulter assay). The experimental setup is illustrated in Fig 8.

Female swine (30–50 kg) were premedicated with intramuscular injections of telazol (~4.4 mg/kg) and xylazine (~4.4 mg/kg). Animals were placed on a surgical table and then intubated with a cuffed endotracheal tube and anesthetized with isofluorane (1–5% inhalation). The anterior and posterior areas of the thorax were shaved for placement of defibrillation electrodes (QUICK-COMBO^TM^ Model 11996; Physio-Control, Redmond, WA with an active area of 115 cm^2^). A vein in the ear was isolated and a catheter was placed for saline administration to keep the animals hydrated. An ECG was recorded using commercially available equipment. Animals were maintained at a temperature of 37 ± 3°C throughout the experiment. An oscilloscope with a voltage and current monitor was used to record each shock.

**Fig 9:**
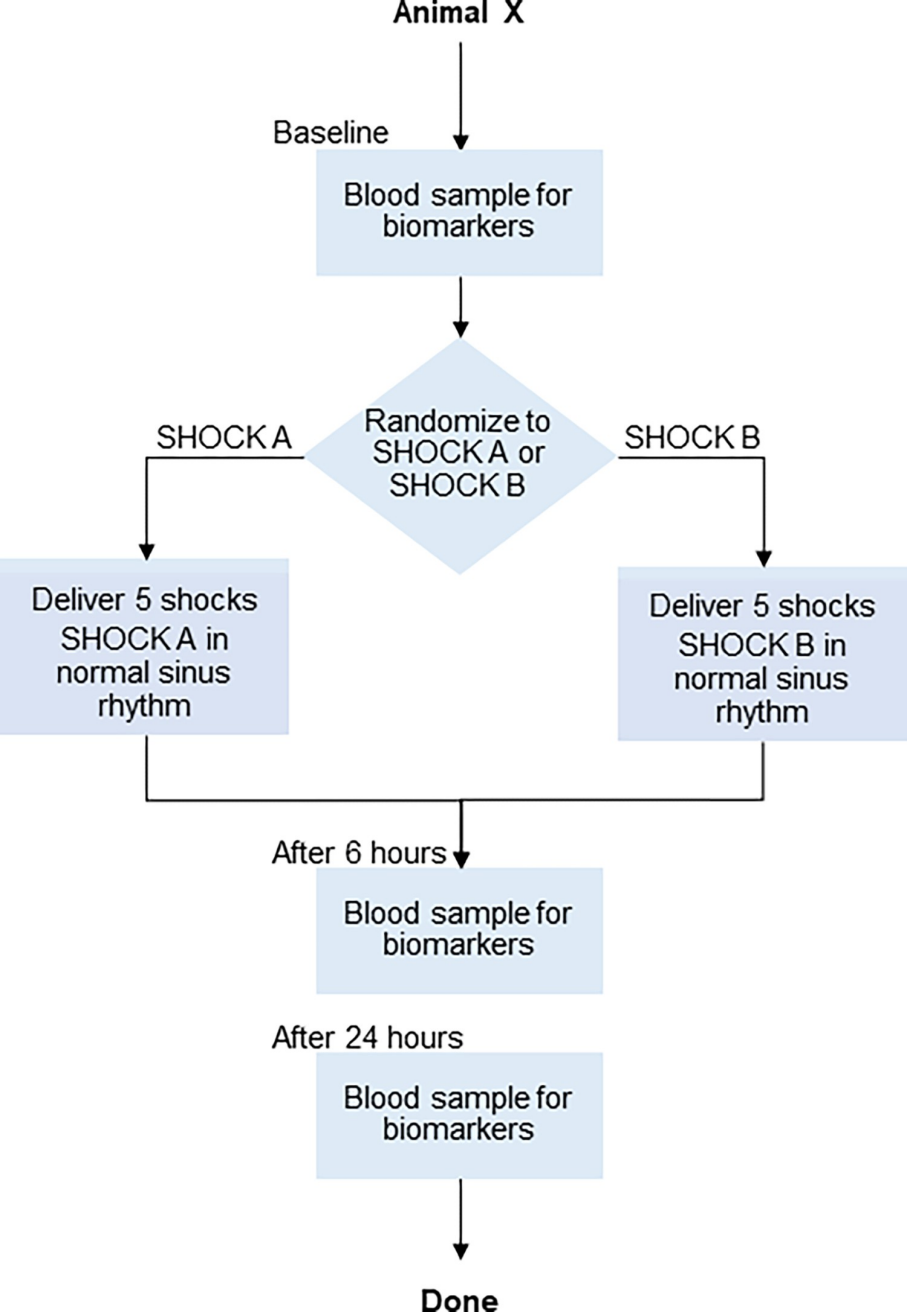
Animal Study #3 shock sequence. Figure illustrating shock sequence and randomization of shock type for each animal.

For each animal, a baseline blood sample was taken prior to the delivery of any shocks. Animals were randomized to receive either SHOCK A five times or SHOCK B five times (Fig 9). Shocks were delivered during normal sinus rhythm to avoid potential impact of ventricular arrhythmias on biomarker levels. Shocks were separated by approximately 30 seconds. Blood samples were drawn again 6 and 24 hours after delivery of the five shocks.

Statistical analysis for Animal Study #3: The primary objective was to compare the change in biomarker levels between animals that received SHOCK A and those that received SHOCK B using measurements obtained at baseline, 6 hours post-shock, and 24 hours post-shock. For each biomarker, the two groups were compared using a Generalized Linear Mixed Model (GLMM). Before GLMM modeling, distribution of each biomarker was assessed for normal distribution by Shapiro-Wilk Test. Non-conformity to the distribution was assumed if the p-value was <0.05. As normality of the response variable was not achieved, Goodness-of-Fit Tests for log-normal distribution were performed. The GLMM was adjusted for log-normal distribution with identity link function and unequal variances (Satterthwaite degree of freedom correction). Changes in biomarkers were evaluated using time interval as the fixed *within*-subject main factor, SHOCK A versus SHOCK B as the fixed *between*-subject main factor, and time interval as the random effects factor at subject level. Final GLMM residuals diagnostics for homoscedasticity and normality were visually performed via studentized residuals plots. The difference in the change in biomarker levels between SHOCK A and SHOCK B was considered significant if the p-value for the type of shock versus time interval interaction was less than 0.05. To correct for Type I error inflation, the Tukey-Kramer post hoc test for pairwise comparisons between SHOCK A and SHOCK B at each time point was performed. Differences were considered significant if the p-value was less than 0.05. The targeted sample size of ten animals was consistent with a similar previously published study [26].

### Human study

After the completion of animal studies, SHOCK A (170J) efficacy and safety were evaluated in adult patients undergoing VF induction in the electrophysiology laboratory in a study called ASSURE WCD Clinical Evaluation–Conversion Efficacy Study (ACE-CONVERT). This was a prospective non-randomized single arm study conducted in the United States at two sites under an investigational device exemption (IDE G190232, NCT04132466). Eligibility criteria are listed in Table 2.

**Table 2:** Human study eligibility criteria.

Inclusion Criteria	Exclusion Criteria
1. Males or females, age ≥ 18 years 2. Able and willing to provide written informed consent before undergoing any study-related procedures 3. Scheduled for any of the following procedures: • Electrophysiology study for induction of ventricular arrhythmias • Non-invasive electrophysiology testing using an existing implantable defibrillator • Implantable cardioverter defibrillator (ICD) replacement procedure during which induction of a ventricular arrhythmia is planned • Ablation of ventricular tachycardia (patients undergoing ventricular tachycardia ablation in which ONLY a substrate modification approach is planned, with no intention of inducing a ventricular arrhythmia, should not be included)	1. Any condition that by the judgement of the physician investigator precludes the subject’s ability to comply with the study requirements 2. Pregnancy 3. Use of any mechanical circulatory support 4. Documented acute cardiac thrombus 5. Atrial fibrillation or atrial flutter without therapeutic systemic anticoagulation 6. Critical aortic stenosis 7. Unstable coronary artery disease 8. Recent stroke or transient ischemic attack 9. Hemodynamic instability 10. Currently implanted entirely subcutaneous ICD 11. Unstable angina 12. New York Heart Association (NYHA) Class IV 13. Left Ventricular Ejection Fraction (LVEF) < 20% 14. Any medical condition that by the judgement of the physician investigator, patient participation in this study is not in the best interest of the patient. 15.History of difficulty of ventricular arrhythmia induction 16. Amiodarone use within 3 months before the study procedure

Patients with known comorbidities associated with increased risk for defibrillation testing were excluded in accordance with published recommendations [27]. The study was conducted in accordance with the International Conference on Harmonisation guideline E6 (R1): Good Clinical Practice: Consolidated Guideline and the principles of the World Medical Association Declaration of Helsinki on Ethical Principles for Medical Research Involving Human Subjects 1964, including all amendments and Notes of Clarification. The study protocol was reviewed and approved for Washington University in St Louis by the Western Institutional Review Board (IRB Tracking Number 20190928, approval 10/22/2019), and for University of Michigan by the University of Michigan Institutional Review Board (ID HUM00164517, approval 10/31/2019). Patients provided written informed consent in accordance with 21 CFR Part 50. Individual subject participation was during acute intra-procedural testing only.

**Fig 10:**
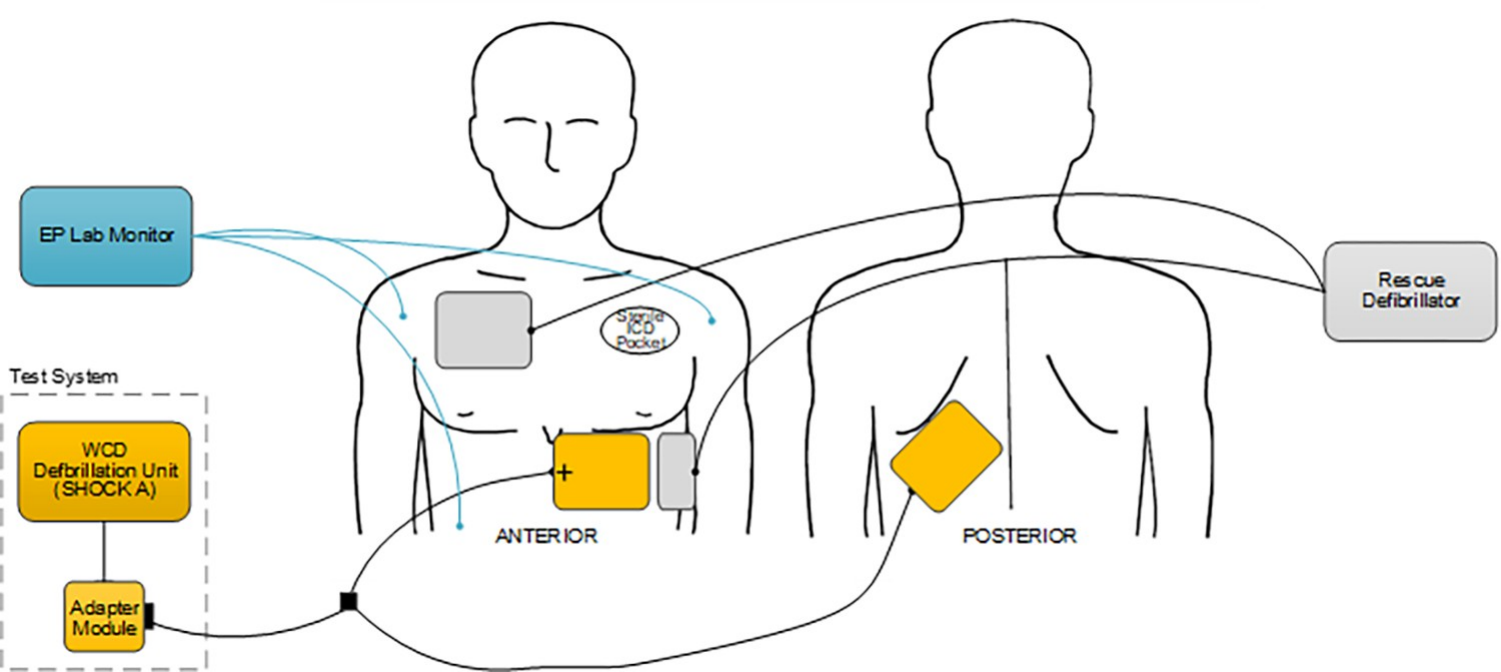
Human study experimental setup. Anterior and posterior views of the thorax for the human study of the 170J BTE waveform (SHOCK A) in the electrophysiology laboratory. The test system showing the WCD defibrillation unit and adapter module is shown in the lower left. Two sets of defibrillation patches are shown, with the test system connected to the yellow patches and rescue defibrillator connected to the gray patches. The test system pads (yellow) were applied in standard anterior and posterior positions, with the anterior pad in the left parasternal region and the posterior pad in the left infrascapular position. The rescue defibrillation pads (gray) were placed in the standard anterior-apical position on the subject’s torso for defibrillation from a commercially available external defibrillator. Surface ECG was monitored with electrophysiology laboratory recording equipment. WCD = wearable cardioverter defibrillator; ICD = implantable cardioverter defibrillator; EP = Electrophysiology.

The investigational set-up is illustrated in Fig 10. The test system included the ASSURE WCD defibrillation unit that delivered SHOCK A with an adapter module that allowed connection to commercially available disposable adhesive defibrillation pads (QUICK-COMBO^TM^ Model 11996; Physio-Control, Redmond, WA). Each pad has an active area of 115 cm^2^. These pads were applied in anterior and posterior positions with the anterior pad in the left parasternal region and the posterior pad in the left infrascapular position. This anterior-posterior position approximates the WCD garment pad locations. Another set of adhesive defibrillation pads were placed in the standard anterior-apical positions on the subject’s torso for rescue defibrillation from a commercially available external defibrillator if required. Surface ECG was monitored using electrophysiology laboratory recording equipment. VF could be induced via rapid pacing or T-wave shocks delivered from the ICD. Once the physician verified VF for at least 5 seconds, he/she directed a technician to initiate SHOCK A, accomplished using a tablet device set up to control the defibrillation unit. If the first SHOCK A was unsuccessful, a second SHOCK A was delivered. If this second SHOCK A was unsuccessful, the commercial external defibrillator was to be used for rescue defibrillation. Energy for rescue defibrillation was at the discretion of the physician investigator. VF duration was measured using electronic or manual calipers on the ECG strip. VF termination was defined as the return of a supraventricular or ventricular QRS complex immediately after shock delivery. Delivered energy and shock impedance were automatically stored by the study device. These measures were then recorded by study personnel on study case report forms. Adverse events were collected at the end of the procedure and followed in person or via telephone until resolution. The primary endpoint was cumulative first and second shock VF conversion rate. Secondary outcome measures included the first shock conversion rate and a summary of adverse events. Detailed definitions of adverse events are provided in the supporting information (S1 File). Demographics, clinical and electrophysiology procedural data were entered in a 21 Code of Federal Regulations Part 11 compliant electronic data capture system.

### Statistical analysis for the human study

The Intention-to-Treat cohort included all subjects who signed informed consent and for whom the study procedure was initiated. The Per-Protocol cohort included all subjects who met all eligibility criteria, signed informed consent, and for whom the procedure was completed without deviation. The primary endpoint was calculated as the ratio of the number of patients with successful first or second shock VF conversion using SHOCK A to the number of total inductions with SHOCK A delivered. Shock success was defined as termination of VF. The success criterion for the primary endpoint was a point estimate of 94% based on the published all-shock conversion rate for the first commercially available WCD [9].

A sample size of 20 patients was chosen to obtain human clinical experience in a comparable number of treated patients as reported in prior series [10, 28]. The sample size precluded analysis of sex-based differences.

Point estimates were presented as number and percentage for categorical data and mean and standard deviation for continuous variables. Confidence intervals (95%) for efficacy endpoints were obtained by binomial exact tests and presented for descriptive purposes only. Data analysis was performed using SAS version 9.4 (SAS Institute, Cary NC).

## Results

### Animal Study #1

A total of 120 VF inductions were performed in six swine (Snyder Farms, AL; 3 female, 3 male; weight range 27.0–33.6 kg, approximate age 9–10 weeks). The first shock success rate for both SHOCK A and SHOCK B was 100%. The first shock success rate of SHOCK A was non-inferior to SHOCK B as the entire 95% confidence interval for the difference was above the -10% non-inferiority margin (p < .001).

### Animal Study #2

**Fig 11:**
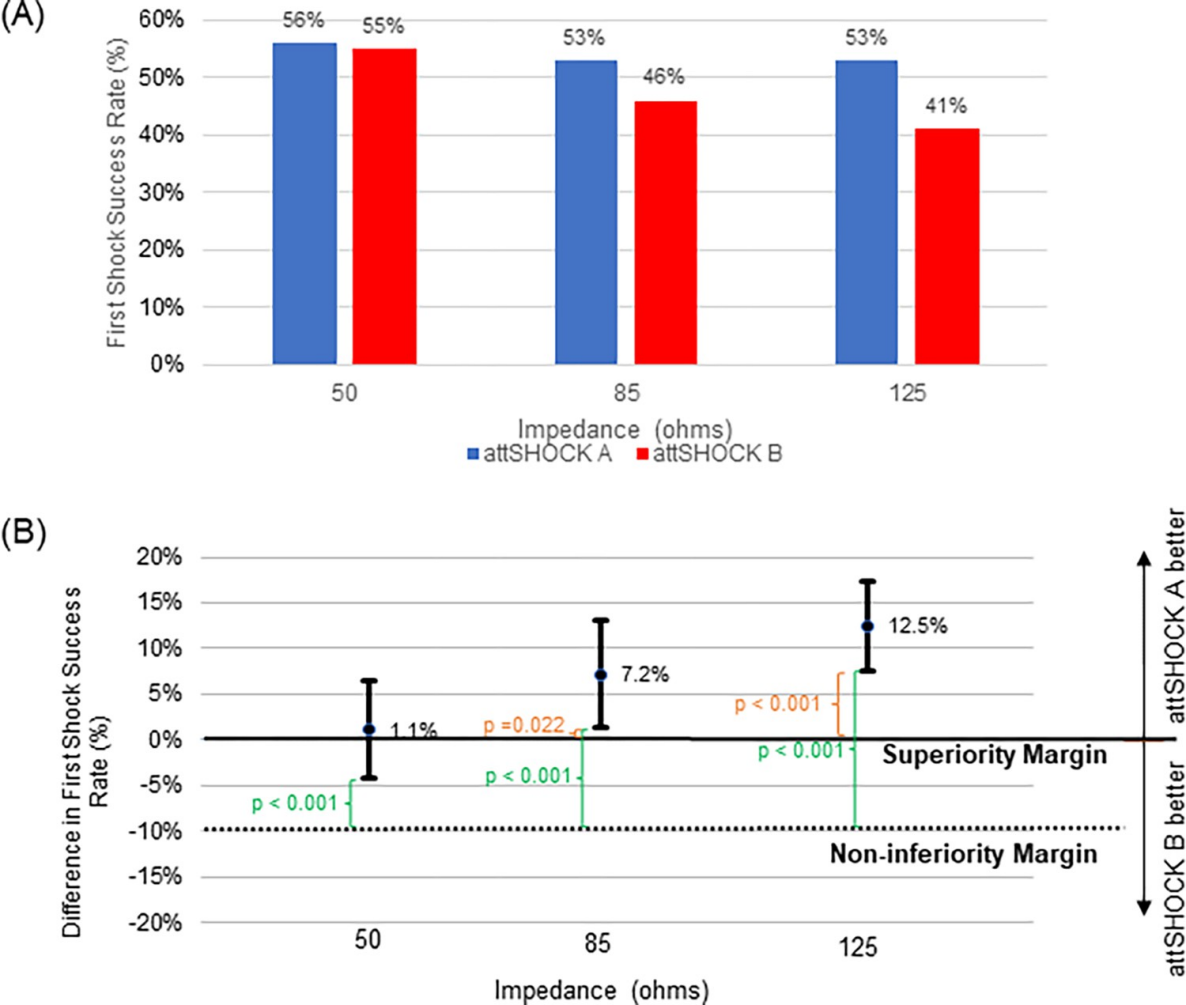
Animal Study #2 results. (A) Observed first shock success rates for termination of ventricular fibrillation with attSHOCK A (blue) and attSHOCK B (red) at 50, 85 and 125 ohm impedances. (B) Difference in observed first shock success rates (attSHOCK A–attSHOCK B). At each of the three impedance levels, the mean difference (dot) and the 95% confidence interval of the difference (whiskers) in first shock success rates is shown. attSHOCK A was non-inferior to attSHOCK B as the entire 95% confidence interval of the difference (attSHOCK A–attSHOCK B) in first shock success rates was above the -10% prespecified non-inferiority margin (dotted line). At 85 and 125 ohms, the first shock success rate of attSHOCK A was superior to attSHOCK B as the entire 95% confidence interval of the difference in first shock success rates was above 0%. See text for details.

A total of 2,160 VF inductions were performed in 36 swine (35 from Prestage Farms, MS and 1 from Snyder Farms, AL; 29 female and 7 male; weight range 7.8–16.6 kg, approximate age 4–7 weeks) to compare shock success rates between attSHOCK A and attSHOCK B at 50, 85, and 125 ohms. Observed shock success rates are shown in Fig 11A. At each of the three impedance levels, the first shock success rate of attSHOCK A was non-inferior to attSHOCK B (Fig 11B) as the entire 95% confidence interval for the difference was above the -10% non-inferiority margin (p < .001). At 85 and 125 ohms, the first shock success rate of attSHOCK A was deemed superior to attSHOCK B as the entire 95% confidence interval was above 0% (at 85 ohms p = .022, at 125 ohms p < .001).

### Animal Study #3

A total of 10 swine underwent biomarker measurement before and after shock delivery (Prestage Farms, MS, all female; weight range 30–50 kg, approximate age 12 weeks). Means and standard deviations of each biomarker level at each time interval are shown in Table 3.

**Table 3:** Animal Study #3 biomarker levels at baseline, 6 hours, and 24 hrs post shock with Tukey-Kramer pairwise comparisons at each time point.

	SHOCK A (n = 5)		SHOCK B (n = 5)		p-values for pairwise comparison between SHOCK A and SHOCK B	
	CPK-MB (IU/L)	Troponin I (ng/mL)	CPK-MB (IU/L)	Troponin I (ng/mL)	CPK-MB	Troponin I
Baseline (mean ± SD)	23 ± 29	0.05 ± 0.05	50 ± 42	0.04 ± 0.06	p = 0.85	p = 0.99
6 hrs (mean ± SD)	289 ± 377	0.16 ± 0.06	723 ± 1368	0.09 ± 0.11	p = 0.99	p = 0.42
24 hrs (mean ± SD)	203 ± 195	0.13 ± 0.09	230 ± 102	0.09 ± 0.04	p = 0.94	p = 0.97

CPK-MB = creatine phosphokinase

The General Mixed Linear Model (GLMM) analysis showed increases in both CPK-MB [F(2, 19.0) = 15.02; p < .001] and troponin I [F(2, 13.6) = 5.76; p = 0.015] using post shock time as the main factor. However, no significant differences were observed for either CPK-MB [F(1, 18.27) = 1.39; p = 0.254] or troponin I [F(1, 20.94) = 3.12; p = 0.092] using type of shock (A versus B) as the main factor, and no significant difference was observed for either CPK-MB [F(2, 19.01) = 0.16; p = 0.855] or troponin I [F(2, 13.6) = 0.43; p = 0.658] when applying the type of shock versus time interval interaction (Table 4). GLMM estimates for the fixed and random effects model are provided as supporting information (S2 File). Tukey-Kramer post hoc pairwise comparisons between animals receiving SHOCK A and those receiving SHOCK B for both CPK-MB and troponin I levels showed no significant difference at each time point (Table 3).

**Table 4:** General Linear Mixed Model analysis for Animal Study #3 biomarker levels.

GLMM Statistics	CPK-MB (IU/L)				Troponin I (ng/mL)			
	df_num_	df_den_	F statistic	p-value	df_num_	df_den_	F statistic	p-value
Time interval main factor	2	19.01	15.02	< .001	2	13.6	5.76	0.015
Type of shock main factor	1	18.27	1.39	0.254	1	20.94	3.12	0.092
Type of shock *versus* Time interval interaction	2	19.01	0.16	0.855	2	13.6	0.43	0.658

GLMM: Generalized Linear Mixed Model; df_num_: Numerator Degree of Freedom; df_den_: Denominator Degree of Freedom; F statistics: Fisher’s distribution statistics.

### Human study

**Fig 12:**
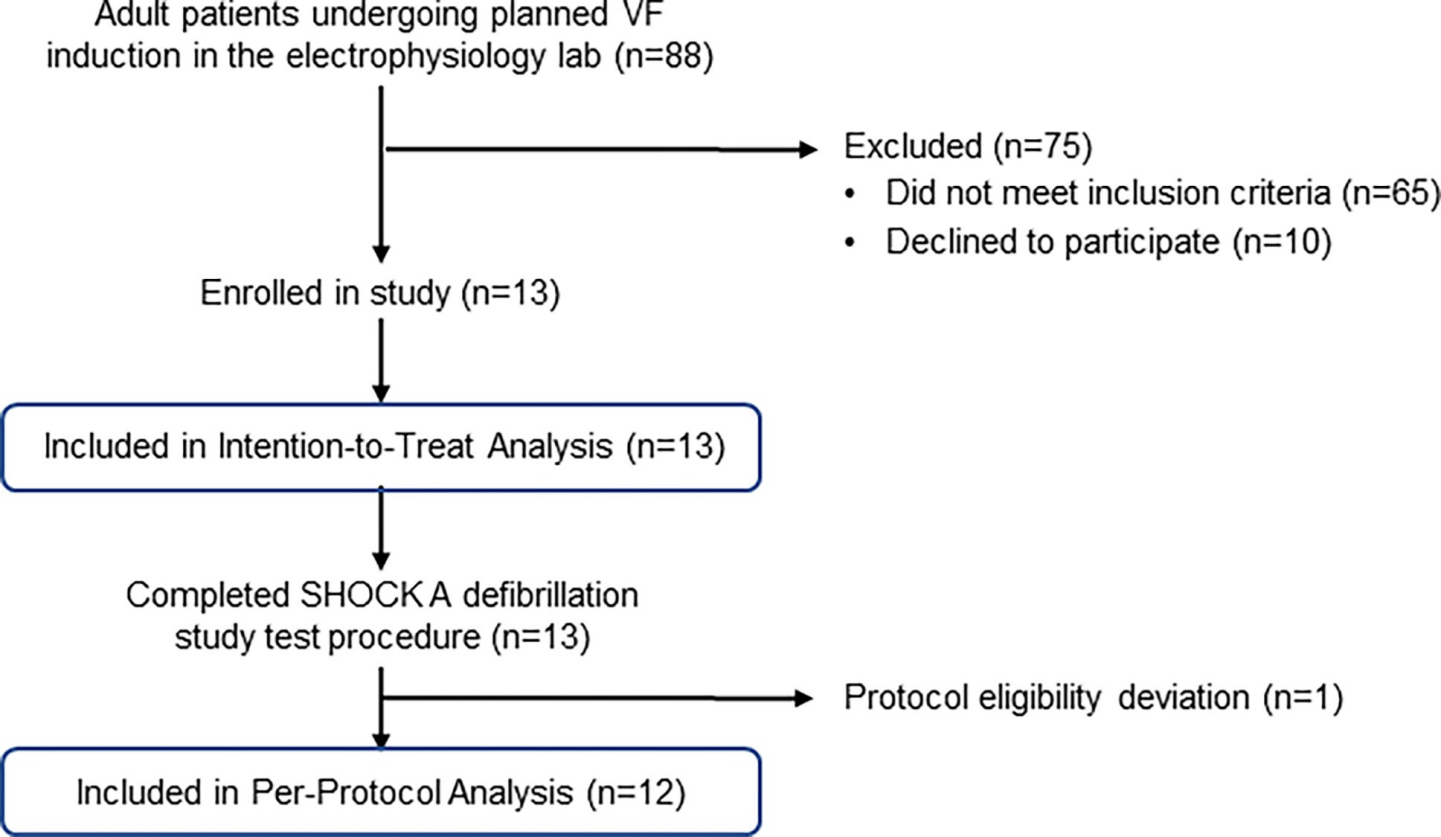
Human study consort flowchart. Of the 88 patients assessed for eligibility, 13 met inclusion criteria and consented to enroll in the study. Defibrillation study procedure was completed on all 13 enrolled patients and included in the Intention-to-Treat analysis. Study monitoring identified that an enrolled patient had been prescribed Amiodarone during an emergency room visit (three weeks prior to study enrollment) and continued taking it for 6 days (protocol eligibility deviation). Twelve patients were included in the Per-Protocol analysis.

Thirteen (13) patients were enrolled at two investigational sites between November 25, 2019, and March 19, 2020 (Fig 12). The sponsor stopped enrollment in March 2020 due to the COVID-19 pandemic in accordance with guidance issued by the United States Food and Drug Administration [29]. Thirteen subjects completed participation by that time. All patients underwent new ICD implant, revision, or replacement with planned induction of ventricular fibrillation. Baseline characteristics are shown in Table 5. Patients had various etiologies of cardiovascular disease and all had comorbidities. Mean left ventricular ejection fraction (LVEF) was 46.8 ± 11.7%. The mean body mass index was 31.5 kg/m^2^ (range 23.7–46.1 kg/m^2^). None of the patients were taking antiarrhythmic drugs at the time of enrollment.

**Table 5:** Human study baseline characteristics.

Characteristic	Value (n = 13)
**Demographics**	
Age (years), mean ± SD	55.3 ± 11.3
Female sex, n (%)	6 (46)
Weight (kg), mean ± SD	88.5 ± 22.4
Body mass index (range)	31.5 (23.7 to 46.1)
Race, n (%)	
Non-Hispanic	0 (0)
Caucasian	10 (77)
Black	3 (23)
**Medical History**	
Etiology of Cardiovascular Disease, n (%)	
Ischemic	2 (15)
Non-ischemic	4 (31)
Congenital/Inherited	4 (31)
Sarcoidosis	3 (23)
Left ventricular ejection fraction %, mean ± SD	46.8 ± 11.7
Abnormal right ventricular function, n (%)	5 (39)
History of heart failure, n (%)	11 (85)
NYHA Functional Classification in patients with heart failure, n (%)	
Class I	1 (8)
Class II	7 (54)
Class III	3 (23)
Comorbidities^a^, n (%)	
Diabetes mellitus	4 (31)
Hypertension	9 (69)
Hyperlipidemia	7 (54)
Prior stroke or transient ischemic attack	1 (8)
Chronic obstructive pulmonary disease	4 (31)
Current smoker	3 (23)
Indication for Initial ICD	
Primary Prevention	10 (77)
Secondary Prevention	3 (23)
ICD Type	
Single chamber	2 (15)
Dual chamber	8 (62)
Cardiac resynchronization defibrillator	3 (23)
**Medication**	
ACE inhibitor	5 (39)
Angiotensin receptor blocker	3 (23)
Beta-blocker	11 (85)
Aldosterone antagonist	4(31)
Angiotensin-neprolysin inhibitor	2 (15)
Digoxin	1 (8)
Antiarrhythmic drug, any	0 (0)

^a^Categories not mutually exclusive

Data are mean ± SD, (N) [min, max], or % (n/N).

NYHA = New York Heart Association

VF was induced via the ICD in all patients. The defibrillation test results are reported in Table 6. Mean time in VF was 14.6 ± 6.7 seconds. A total of 15 shocks (SHOCK A) were delivered to 13 patients. For the 15 shocks delivered, the mean transthoracic impedance (TTI) was 79.6 ± 21.6 ohms (range 50–126). Mean energy delivered was 162.0 ± 7.1J. Two patients required a second shock for successful termination of VF. In the first of these two patients, the TTI was 82 ohms for the first shock and 81 ohms for the second shock. In this patient the delivered energy was 165J for both shocks and the total time in VF was 22.1 seconds. The second patient had a TTI of 126 ohms for the first shock and 124 ohms for the second shock. In this patient the delivered energy was 145J for both shocks and total time in VF was 16.9 seconds. No patient required rescue defibrillation nor required any intervention for hemodynamic or rhythm instability.

**Table 6:** Human study defibrillation test results.

Patient	Induced Rhythm	First Shock			Second shock			Time in VF (seconds)
		Impedance (ohms)	Delivered Energy (joules)	Converted (Yes/No)	Impedance (ohms)	Delivered Energy (joules)	Converted (Yes/No)	
1	VF^b^	81	165	Yes	-	-	-	12.6
2	VF^b^	59	166	Yes	-	-	-	13.5
3	VF^b^	91	162	Yes	-	-	-	16.2
4	VF^b^	82	165	No	81	165	Yes	22.1
5	VF^b^	126	145	No	124	145	Yes	16.9
6	VF^b^	70	164	Yes	-	-	-	5.0
7^a^	VF^b^	81	165	Yes	-	-	-	8.4
8	VF^b^	67	164	Yes	-	-	-	12.3
9	VF^c^	70	165	Yes	-	-	-	15.2
10	VF^b^	63	165	Yes	-	-	-	8.3
11	VF^c^	62	166	Yes	-	-	-	27.7
12	VF^c^	87	164	Yes	-	-	-	23.8
13	VF^b^	50	168	Yes	-	-	-	8.0

^a^Subject excluded from the Per-Protocol analysis

^b^VF induction via T-wave shock

^c^VF induction via rapid pacing

The primary endpoint using both Intention-to-Treat and Per-Protocol analyses was met. The point estimate of cumulative first and second shock efficacy for conversion of VF was 100%, exceeding the 94% pre-specified performance goal (Table 7). The secondary endpoint, first shock VF conversion efficacy, was 84.6% and 83.3% in the Intention-to-Treat and Per-Protocol analyses, respectively. Three adverse events occurred in three subjects (23%). All three events were mild skin irritation from the adhesive defibrillation pads and all resolved within 12 days (range 3–12, mean 8 days).

**Table 7:** Human study defibrillation efficacy.

	Intention-to-Treat Analysis (n = 13)	Per-Protocol Analysis (n = 12)
Primary endpoint:	13 out of 13	12 out of 12
Cumulative first and second shock VF conversion efficacy	100.0% (95% CI: 75.3%, 100.0%)	100.0% (95% CI: 73.5%, 100.0%)
Secondary endpoint:	11 out of 13	10 out of 12
First shock VF conversion efficacy	84.6% (95% CI: 54.6%, 98.1%)	83.3% (95% CI: 51.6%, 97.9%)

VF = ventricular fibrillation; CI = confidence interval

## Discussion

In this manuscript, we report the results of three animal studies and one human study. Animal Study #1 provides comparative evidence of defibrillation efficacy of the shock waveform used in the recently approved WCD (SHOCK A) to a shock waveform that approximates that of another commercially available BTE waveform (SHOCK B). As the commercially available devices have different waveform parameters and different nominal energies, a demonstration of defibrillation efficacy is clinically relevant to prescribing physicians. Animal Study #2 provides a novel approach for comparison of defibrillation efficacy as it utilized proportionally attenuated charge voltages (attSHOCK A and attSHOCK B). Animal Study #2 also evaluated defibrillation efficacy across a range of clinically relevant impedances. Animal Study #3 provides comparative safety evidence between SHOCK A and SHOCK B by measuring changes in biomarker levels following shock delivery. The human study provides a point-estimate of SHOCK A success for terminating induced VF. The key findings are 1) SHOCK A and SHOCK B were both effective at terminating VF in animals; 2) at high transthoracic impedances attSHOCK A was superior at terminating VF in animals compared to attSHOCK B; 3) no statistically significant difference was seen in the change in biomarker levels between SHOCK A and SHOCK B in animals; and 4) SHOCK A was successful in converting induced VF in humans.

Previous studies have used various methodologies to evaluate defibrillation efficacy in animals. One methodology is to compare the efficacy of shocks available from different external defibrillators [17]. This approach was applied in Animal Study #1. However, BTE waveforms are highly effective (> 80% first shock success) and this methodology may not identify potential efficacy differences. Another methodology is defibrillation threshold (DFT) testing [22, 30]. While this approach establishes the *energy* required for 50% defibrillation success, it does not predict the *relative shock success rates* between two clinical devices because dose-response curves are non-linear [31]. We therefore used a novel approach in Animal Study #2 that addressed these drawbacks by measuring shock success rates at proportionally reduced (attenuated) charge voltages. SHOCK A charge voltage was reduced to a level that achieved approximately 50% success and then SHOCK B charge voltage was reduced by the same percentage. By comparing shock success rates at these reduced levels, we were able to observe success rate differences at high impedance levels that were not observed in Animal Study #1.

Transthoracic impedance (TTI) in humans can vary widely based on several factors as described in a recent meta-analysis [32]. The factors that most strongly influence TTI when using adhesive pads are electrode size and position. The mean TTIs reported in the meta-analysis studies varied from 41 to 128 ohms with an overall mean of 77.8 ± 16.4 ohms. In our human study, electrode size and position were controlled and the mean TTI was 79.6 ± 21.6 ohms (50–126 ohms).

Recognizing that TTI varies in humans, we designed Animal Study #2 to evaluate defibrillation efficacy across the expected range of human impedance. This study demonstrated that shock success for attSHOCK A was non-inferior to attSHOCK B at 50, 85, and 125 ohms. In addition, statistical superiority for attSHOCK A was demonstrated at 85 and 125 ohms. This suggests that efficacy is similar at low impedance, however differences might be observed clinically in high impedance patients.

The higher energy of SHOCK A (170J) compared to SHOCK B (150J) raises the question of potentially higher myocardial injury. Animal Study #3 was designed to address this by assessing cardiac biomarker levels post-shock. For both shocks, while troponin I and CPK-MB levels increased from baseline, the increases between the two shocks were not significant. These data suggest SHOCK A does not pose a higher risk of myocardial injury than SHOCK B in animals.

The three animal studies provided statistically significant evidence of safety and effectiveness of the new WCD waveform in terminating VF. While evidence of human defibrillation efficacy is necessary, obtaining this data poses inherent risk. Our series of animal studies along with a limited human study was intentionally designed to minimize the risk to humans. The cumulative first and second shock success rate for conversion of induced VF in our human study was 100%. The first shock success rate was 84.6% in the intention-to-treat analysis and 83.3% in the per-protocol analysis. The only comparable results were reported by Reek et al. [28]. In that study, which was also performed the EP laboratory, 12 patients had VF induced with a 100% first shock success using the other commercially available WCD. However, the shock success rate at higher impedance values is unknown as the highest TTI reported was 79 ohms. First shock success rates of spontaneous VT/VF have been reported in registry studies for the first commercially available WCD [9–11, 33]. In these studies, the first shock success rates ranged between 84% and 100%, however definitions of episodes varied. The shock success rate observed in our human study is consistent with these prior reports.

## Limitations

### Human study

Due to COVID-19 restrictions, testing of SHOCK A was halted after 13 patients completed the study instead of the 20 that were originally planned. However, the number of patients receiving a shock (13) is comparable to prior published series of WCD patients. Defibrillation was performed using commercially available adhesive pads because the sterile field precluded use of the WCD garment. VF was induced rather than spontaneous and while there are recognized differences, there is no evidence that these differences impact shock efficacy [34]. Time in VF for all cases was shorter than time to shock for a WCD in clinical use. For patient safety, minimizing time in VF was a necessary condition for conducting a defibrillation study in the electrophysiology laboratory setting. Additionally, the study population may not be representative of the broader WCD population.

### Animal studies

Waveform generators were designed to deliver SHOCK A and SHOCK B, replicating two commercially available therapies. In Animal Study #2 in small swine, resistors were used to simulate a range of impedances. It is possible that the current distribution in the thorax may have been different if the swine had been larger or had naturally exhibited a wider range of impedances. As this was a comparative study, we would anticipate that the current distribution in each animal would be similar for attSHOCK A and attSHOCK B. Furthermore, Animal Study #1 in large swine in the absence of additional resistors demonstrated 100% first shock success for both SHOCK A and SHOCK B.

## Conclusions

The BTE defibrillation shock waveform used in the recently approved WCD was highly effective at terminating induced VF in animals. Success was also demonstrated in a small sample of humans. Safety analyses did not indicate any significant or unanticipated clinical complications. Future studies in patients at high risk of sudden cardiac death wearing this WCD may provide further evidence of defibrillation efficacy and safety.

## Supporting information

S1 File Adverse event definitions. (PDF)

S2 File GLMM estimates for fixed and random effects model for Animal Study #3. (PDF)

S3 File TREND statement checklist. (PDF)

S4 File ACE-CONVERT clinical trial plan. (PDF)
