# Supplementary material for: Defibrillation effectiveness and safety of the shock waveform used in a contemporary wearable cardioverter defibrillator: Results from animal and human studies
Source: PLoS One. 2023 Mar 14;18(3):e0281340. doi: 10.1371/journal.pone.0281340 (PMC10013906; doi:10.1371/journal.pone.0281340)
Supplement: S1 File — (PDF) [file pone.0281340.s001.pdf]

Adverse event definitions

Adverse events (AEs) were defined as any untoward medical occurrence in a subject during the study that in the opinion of the investigator were at least possibly related to use of the Test System. An independent physician medical monitor adjudicated all AEs, assessing seriousness, severity, and relatedness to the study device, and further determined whether any were reportable as unanticipated adverse device effects (UADEs).

The Medical Monitor classified the severity of each adverse event using the following definitions:

Mild: Awareness of signs and symptoms, but easily tolerated; are of minor irritant type, causing no loss of time from normal activities; symptoms would not require medication or a medical evaluation; signs and symptoms are transient.

Moderate: Discomfort severe enough to cause interference with usual activities; requiring treatment, but not extended hospitalization or intensive care for the subject.

Severe: Incapacitating with inability to do work or usual activities; signs and symptoms may be systemic in nature or require medical evaluation and/or treatment; requiring additional hospitalization or intensive care (prolonged hospitalization).

A Serious Adverse Event (SAE) was defined as any adverse event that led to death or serious deterioration in the health of a subject that resulted in a life-threatening illness or injury; resulted in a permanent impairment of a body structure or a body function; required an inpatient or prolonged hospitalization ≥ 24 hours; resulted in medical or surgical intervention to prevent life-threatening illness or injury or permanent impairment to a body structure or function; or led to fetal distress, fetal death or a congenital anomaly or birth defect. Because pregnancy is an exclusion criterion and confirmation of a negative pregnancy test for females of childbearing potential was recorded, a data field to record an SAE result of fetal distress, fetal death or a congenital anomaly or birth defect was not included. AEs that persisted at the time of the subject’s study exit were followed by the investigator until the event was resolved, the subject was lost to follow-up, or the adverse event was otherwise explained.
