# Supplementary material for: Defibrillation effectiveness and safety of the shock waveform used in a contemporary wearable cardioverter defibrillator: Results from animal and human studies
Source: PLoS One. 2023 Mar 14;18(3):e0281340. doi: 10.1371/journal.pone.0281340 (PMC10013906; doi:10.1371/journal.pone.0281340)
Supplement: S2 File — (PDF) [file pone.0281340.s002.pdf]

GLMM estimates for fixed and random effects model for Animal Study #3

	CPK-MB (IU/L)		Troponin I (ng/mL)	
	**Estimate**	**Standard Error**	**Estimate**	**Standard Error**
Fixed Effects (Coefficients)				
Intercept	3.4935	0.4698	-3.7555	0.4566
Shock B	0	-	0	-
Shock A	-0.7661	0.6643	0.3800	0.6457
Baseline	0	-	0	-
6h	1.8286	0.7905	1.0069	0.5528
24h	1.8804	0.5501	1.1963	0.5557
Shock B *versus* Baseline	0	-	0	-
Shock A *versus* Baseline	0	-	0	-
Shock B *versus* 6h	0	-	0	-
Shock A *versus* 6h	0.4899	1.1179	0.4992	0.7817
Shock B *versus* 24h	0	-	0	-
Shock A *versus* 24h	0.4090	0.7780	-0.04887	0.7859
Random Effects (Covariance)				
Time interval (*subject level*)	0.637	0.340	0.273	0.195
Residual	0.541	-	0.404	-
