# Supplementary material for: Defibrillation effectiveness and safety of the shock waveform used in a contemporary wearable cardioverter defibrillator: Results from animal and human studies
Source: PLoS One. 2023 Mar 14;18(3):e0281340. doi: 10.1371/journal.pone.0281340 (PMC10013906; doi:10.1371/journal.pone.0281340)
Supplement: S4 File — (PDF) [file pone.0281340.s004.pdf]

**PROTOCOL TITLE:**	ASSURE WCD Clinical Evaluation – Conversion Efficacy Study (ACE-CONVERT)
**PROTOCOL NUMBER:**	3333934_C
**INVESTIGATIONAL THERAPY:**	ASSURE WCD 170 Joule biphasic defibrillation waveform
**STUDY DESIGN:**	Multicenter single arm open label evaluation
**SPONSOR:**	Kestra Medical Technologies, Inc.<br>3933 Lake Washington Blvd NE, Suite 200<br>Kirkland, WA 98033
**DATE OF PROTOCOL:**	10/4/2019

This study will be conducted in accordance with the International Conference on Harmonisation guideline E6 (R1): Good Clinical Practice: Consolidated Guideline and the principles of the World Medical Association Declaration of Helsinki on Ethical Principles for Medical Research Involving Human Subjects 1964, including all amendments and Notes of Clarification.

3 PROTOCOL SUMMARY

**Protocol Number:**	3333934_C
**Protocol Title:**	ASSURE WCD Clinical Evaluation – Conversion Efficacy Study (ACE-CONVERT)
**Study Population:**	Patients who are scheduled for any of the procedures specified in Inclusion Criterion 3 (below).
**Study Design:**	Multicenter single arm open label
**Number of Subjects:**	20
**Study Sites:**	Subjects will be enrolled at up to 5 clinical sites in the United States where procedures specified in Inclusion Criterion 3 (below) are routinely performed. There is no minimum or maximum number of subjects that may be enrolled at each site.
**Eligibility Criteria:**	Subjects who meet *all* Inclusion Criteria and *no* Exclusion Criteria may be enrolled in the study.<br><br>**Inclusion Criteria**:<br>1) Males or females, age ≥ 18 years<br>2) Able and willing to provide written informed consent before undergoing any study-related procedures<br>3) Scheduled for any of the following procedures:<br>a) Electrophysiology study for induction of ventricular arrhythmias<br>b) Non-invasive electrophysiology testing using an existing implantable defibrillator<br>c) ICD replacement procedure during which induction of a ventricular arrhythmia is planned<br>d) Ablation of ventricular tachycardia (patients undergoing ventricular tachycardia ablation in which ONLY a substrate modification approach is planned, with no intention of inducing a ventricular arrhythmia, should *not* be included)

	**Exclusion Criteria**:<br>1. Any condition that by the judgement of the physician investigator precludes the subject's ability to comply with the study requirements<br>2. Pregnancy<br>3. Use of mechanical circulatory support (e.g. LVAD, Total Artificial Heart, intraaortic balloon pump or Impella)<br>4. Documented nonchronic cardiac thrombus<br>5. Atrial fibrillation or atrial flutter without therapeutic systemic anticoagulation<br>6. Critical aortic stenosis<br>7. Unstable coronary artery disease (CAD)<br>8. Recent stroke or transient ischemic attack (TIA)<br>9. Hemodynamic instability<br>10. Currently implanted Boston Scientific S-ICD (due to location of implant relative to test system)<br>11. Unstable angina<br>12. New York Heart Association (NYHA) Class IV<br>13. Left Ventricular Ejection Fraction (LVEF) < 20%<br>14. Any medical condition that by the judgement of the physician investigator, patient participation in this study is not in the best interest of the patient<br>15. History of difficulty of ventricular arrhythmia induction<br>16. Amiodarone use within 3 months before the study procedure
**Investigational Therapy:**	ASSURE 170 Joule biphasic defibrillation waveform
**Energy Delivery Protocol:**	Up to two 170 Joule shocks will be delivered from the Test System. If the second shock is unsuccessful, additional rescue shocks will be delivered using a commercially available internal or external defibrillator.
**Study Procedures:**	Cardiac patients will be screened for eligibility by the clinical site staff members in advance of the procedure date. Eligible patients will be provided the informed consent form and will be allowed enough time to review the consent, and have any questions adequately addressed. Only patients who provide written informed consent to participate will be enrolled as study subjects. Conscious sedation or general anesthesia will be administered per investigator preference. Participating subjects will have two sets of commercially available disposable adhesive defibrillation pads applied in advance of the procedure. One set will be placed in defined locations and used to deliver the experimental defibrillation shock using the Test System. The other pair will be located on the subject's torso according to physician preference and will be attached to a commercially available external defibrillator for backup rescue defibrillation. Backup rescue defibrillation can

	also be done using an internal defibrillator per the physician preference. Commercially available ECG monitoring electrodes will be positioned as needed. The ASSURE WCD Monitor will be configured to Manual Shock mode, which allows the experimental shock to be delivered by a Kestra Clinical Specialist on command of the physician Investigator or Sub-Investigator.<br><br>A single sustained episode of rapid VT or VF will be induced during an electrophysiologic study from a catheter or an implanted defibrillator or may occur spontaneously. If the Arrhythmia is > 150bpm, then a 170J shock will be delivered from the Test System to convert the arrhythmia. A second shock at 170J will be delivered from the Test System if the first shock is unsuccessful.<br><br>Further rescue shocks may be delivered via an internal or external defibrillator at the Investigator's discretion.<br><br>Conversion is defined as the presence of a non-shockable rhythm after shock delivery. Documentation that will be collected includes captured screen shots and log files from the Tablet programmer, paper copies of ECG strips from the EP monitoring system, and a study-specific physician procedure report.
**Efficacy Objective:**<br>**Primary Endpoint:**<br>**Secondary Endpoint:**<br><br>**Safety Objective:**<br><br>**Safety Endpoint:**	To evaluate conversion efficacy of the ASSURE WCD Waveform<br>Estimated cumulative first and second shock VT/VF conversion efficacy ≥ 94%<br>First shock VT/VF conversion efficacy (report only)<br><br>To collect information on adverse events at least possibly related to use of the investigational Test System<br><br>Summary of adverse events that are at least possibly related to use of the investigational Test System, including classification of Unanticipated Adverse Device Effects
**Study Duration:**	Individual subject participation is during acute intra-procedural testing only. Subjects can be enrolled only once. Total Study Duration from first subject in to last subject out will be approximately two months.

Table of Contents

1 SPONSOR SIGNATURE PAGE 2
2 KEY ROLES AND CONTACTS 3
3 PROTOCOL SUMMARY 4
4 GLOSSARY OF ABBREVIATIONS AND DEFINITION OF TERMS 11
5 INTRODUCTION 12
Background 12
Rationale for Approach 12
PRECLINICAL STUDIES 14
6 OVERALL STUDY PLAN 17
Number of Subjects 17
Study Sites 17
Study Population 17
Eligibility Criteria 17
Participation Period 18
Subject Withdrawals 18
Medical Monitor 19
Sponsor or Regulatory Agency Termination of the Study 19
End of Study 19
7 STATISTICAL PLAN 20
Data Sources 20
Study Objectives and Endpoints 20
Sample Size Considerations 20
Analysis Populations 20
Handling of Missing Data 21
8 TEST SYSTEM 25
Safety and Device Accountability 28
Reuse of System Components 28
9 STUDY PROCEDURES 29
Informed Consent and Enrollment 29
Assessments 29
Demographic Data 29
Medical History 29
Clinical Laboratory Tests 30
Supply, Packaging, Labeling, and Storage 30
Electrophysiology (EP) Procedure 30
Screen Failures 32
Handling of Withdrawals 32
10 ADVERSE EVENT REPORTING 34
11 DATA HANDLING AND QUALITY ASSURANCE 38
Subject Identification Numbers 38
Case Report Forms 38
Study Data and Conduct Monitoring 38
Inspection of Records 39
Study Record Retention 39
Ethical Conduct of the Study 40
Informed Consent 40
Institutional Review Board 40

12 ADMINISTRATIVE CONSIDERATIONS 41
Confidentiality 41
Modification of the Protocol 41
Protocol Deviations 41
Study Reporting Requirements 42
Financial Disclosure 42
Financial Obligations 42
Investigator Documentation 42
Clinical Trial Agreement 43
Policy for Publication and Presentation of Data 43
13 INVESTIGATOR AGREEMENT 44
14 APPENDIX A - ASSURE DEFIBRILLATION WAVEFORM 45
15 APPENDIX B - MEDICARE IDE STUDY CRITERIA 46
16 BIBLIOGRAPHY 47

LIST OF IN-TEXT FIGURES

Figure 1: Example Test System and Monitoring Setup 26

Figure 2: Test System Defibrillation Pad and ECG Electrode Placement 27

Figure 3: Time to shock 32

Figure 4: Defibrillation Waveform Profiles into 50 ohms 45

LIST OF IN-TEXT TABLES

Table 1: Spontaneous Defibrillation Efficacy Observed in ZOLL LifeVest Registry Studies 13

Table 2: Summary of Reduced Energy Level Study in Swine Results 15

Table 3: Steps to Minimize Missing Data for the Primary Endpoint 21

Table 4: Pass/Fail Criteria - Estimated Conversion Rate Based on Study Results 23

4 GLOSSARY OF ABBREVIATIONS AND DEFINITION OF TERMS

ABBREVIATION	DEFINITION
AADE	Anticipated Adverse Device Effect
ADAM	ASSURE Defibrillation Adapter Module
AE	Adverse Event
AED	Automated External Defibrillator
CAD	Coronary Artery Disease
CFR	Code of Federal Regulations
CIED	Cardiac Implantable Electronic Device
CPVT	Catecholaminergic Polymorphic Ventricular Tachycardia
CRA	Clinical Research Associate
CRF	Case Report Form
CRO	Contract Research Organization
ECG	Electrocardiogram
EPS	Electrophysiology Study
FPI	First Patient In
FDA	Food and Drug Administration
GCP	Good Clinical Practice (Guidelines)
GDR	General Document Record
ICD	Implantable Cardioverter-Defibrillator
ICF	Informed Consent Form
ICH	International Conference on Harmonisation
IEC	International Electrotechnical Commission
ILR	Implantable Loop Recorder
IRB	Institutional Review Board
LPO	Last Patient Out
LVAD	Left Ventricular Assist Device
LVEF	Left Ventricular Ejection Fraction
MI	Myocardial Infarction
PP	Per-Protocol
PEA	Pulseless Electrical Activity
SAE	Serious Adverse Event
SCA	Sudden Cardiac Arrest
S-ICD	Subcutaneous Implantable Cardioverter-Defibrillator
TIA	Transient Ischemic Attack
UADE	Unanticipated Adverse Device Effect
VF	Ventricular Fibrillation
VT	Ventricular Tachycardia
WCD	Wearable Cardioverter Defibrillator

5 INTRODUCTION

Background

The Wearable Cardioverter-Defibrillator (WCD) is indicated for adult patients who are at risk of sudden cardiac arrest (SCA) and are not immediate candidates for or decline an implantable defibrillator. The study Sponsor has developed a new wearable defibrillator, the ASSURE WCD, to address this clinical situation.

The ASSURE WCD uses a biphasic truncated exponential waveform to deliver 170 Joules from a 140µF capacitor. The waveform is like that used by existing commercially available devices, including Physio-Control LifePak12®, the Philips Heartstart® Automated External Defibrillator (AED), and the ZOLL LifeVest® Wearable Cardiac Defibrillator (APPENDIX A).

This study is part of a comprehensive set of evaluation protocols which has been proposed to establish safety and effectiveness of the ASSURE WCD and will be included in support of the Premarket Approval (PMA) application for the ASSURE WCD.

Rationale for Approach

Defibrillation efficacy testing was once considered a standard part of ICD implantation. Decades of clinical experience with biphasic waveforms and other significant improvements in ICD technology over the years raised the question of the value of routine defibrillation testing resulting in the initiation of several large, prospective randomized controlled studies. Based on the results of these studies, the following recommendation was issued in a joint statement by the four continental electrophysiology societies in 2015 (Wilkoff B):

> "*It is **reasonable to omit defibrillation efficacy testing** in patients undergoing initial left pectoral transvenous ICD implantation procedure where appropriate sensing, pacing, and impedance values are obtained with fluoroscopically well-positioned RV lead*s."

As a result, defibrillation threshold testing is less frequently performed, and a strategy which minimizes dependence on human defibrillation testing to establish safety and effectiveness for new devices is necessary.

The Sponsor's strategy includes a comprehensive set of bench and animal tests that have been conventionally used to evaluate defibrillation efficacy of biphasic waveforms in AEDs with characteristics that are proven and well-understood. These bench and animal studies include:

1. Demonstration of fundamental electrode characteristics required for successful defibrillation (**Impedance and active area meet IEC standards and conventional Anterior-Posterior placement**).
2. Assessment of efficacy by a rigorous study of waveform and dose in animals (Non-inferior to the predicate to establish equivalence): Sponsor Protocol 3337920 *Comparing Efficacy of the ASSURE Wearable Automated External Defibrillator (AED) and the ZOLL LifeVest Defibrillation Waveforms*.
3. Assessment of ASSURE full energy waveform: Sponsor Protocol 3338359 *ASSURE Full Energy Shock Efficacy*.
4. Assessment of ASSURE full energy waveform: Sponsor Protocol 3337766 *Waveform Safety Study*.

In addition, this clinical study is being conducted in humans who are undergoing electrophysiologic procedures for ventricular arrhythmias to demonstrate full energy shock conversion in humans.

Recent, prospective published registry studies of the ZOLL LifeVest provide a relevant reference for establishing a point estimate of defibrillation efficacy for this study. Spontaneous defibrillation efficacy observed in these registry studies is summarized in Table 1.

Table 1: Spontaneous Defibrillation Efficacy Observed in ZOLL LifeVest Registry Studies

Registry Study	Number of Subjects	Number of Subjects with Events	Number of Successful Conversions	Conversion Rate
US Registry (WEARIT-II) **(Kutyifa V)**	2,000	22	22	100% (C.I. 0.873 – 1.000)
German Registry **(Wäßnig NK)**	6,043	94	88	94% (C.I. 0.866 – 0.977)

PRECLINICAL STUDIES

The following preclinical studies have been completed to demonstrate safety and effectiveness of the waveform in animals:

Efficacy of the ASSURE and LifeVest Waveforms at Full-Energy in Swine (Protocol 3338359)

This prospective block-randomized study compared the efficacy of the ASSURE defibrillation waveform at *full energy output (170J)* in treating electrically-induced VF in a closed chest porcine model of short duration cardiac arrest in comparison to the ZOLL LifeVest defibrillation waveform at *full energy output (150J)*. Six swine were treated for 20 VF episodes each. The VF episodes were organized in 10 blocks, with each block consisting of one shock from each device in random order. Both devices produced a 100% shock success rate in all six animals. This study was designed to measure the difference in success rate between the two devices. Because there was no variation in the data, the lower-bound 95% confidence interval of the difference in success rates (ASSURE shock success rate – LifeVest shock success rate) between the two devices is 0% which meets the non-inferiority criteria.

Overall, this study provides evidence that the ASSURE WCD waveform is non-inferior to the LifeVest waveform in treating VF with full-energy shocks.

Efficacy of the ASSURE and LifeVest Waveforms at Reduced Energy Levels in Swine (Protocol 3337920)

This prospective block-randomized study compared the efficacy of the ASSURE defibrillation waveform at *attenuated energy output (ED50)* in treating electrically-induced VF in a closed chest porcine model of short duration cardiac arrest compared to the currently marketed ZOLL LifeVest defibrillation waveform *attenuated energy output*. Waveform voltages were attenuated to provide greater sensitivity to detect waveform efficacy differences. Probability of defibrillation shock success was compared at three impedance levels (50, 85, and 125 Ω). Thirty-six (36) animals were studied.

A summary of the protocol that was followed for each animal is as follows: (1) The ASSURE WCD shock voltage was attenuated to the level that produced an approximate 50% shock success rate (ED50); (2) The LifeVest shock voltage was attenuated by the same percentage; (3) With these voltage levels, ten block-randomized shocks were delivered with each waveform and shock successes and failures were recorded; (4) steps 1, 2, and 3 were repeated at each of the three impedance levels (50, 85, and 125Ω); and

(5) The success rate was calculated for each waveform at each impedance level. The lower-bound 95% confidence interval for the difference between the two devices was compared (ASSURE WCD shock success – LifeVest shock success). The results from this calculation are shown in Table 2, below.

Table 2: Summary of Reduced Energy Level Study in Swine Results

Impedance Level	Effect Size	Lower-Bound 95% Confidence Interval	Pass/Fail Limit	Pass or Fail Non-Inferiority Criteria?
50Ω	1.11%	-4.22%	-10%	Pass
85Ω	7.22%	1.38%	-10%	Pass
125Ω	12.50%	7.62%	-10%	Pass

Effect size = ASSURE waveform shock success – LifeVest waveform shock success

Overall, this study provides evidence that the ASSURE WCD waveform is non-inferior to the LifeVest waveform at 50Ω, 85Ω, and 125Ω.

__**Waveform Safety Study in Swine (Protocol 3337766)**__

This study compared the safety of the ASSURE WCD waveform at *full energy output (170J)* in swine to a commercially-available external defibrillator (LIFEPAK 12) waveform at 200J. The following protocol was followed for each animal (N = 8): (1) at baseline, blood was drawn and ECG collection was started; (2) five shocks were delivered from the randomly assigned study device; (3) ECG collection continued until at least 1 hour post-shock; (4) 6 hours post shock, another blood sample was drawn and 10 minutes of ECG collected; (5) at least 24 hours post shock, another blood sample was drawn, animal was euthanized, and tissue samples were collected for histological evaluation; and finally (6) the change in biomarkers from the baseline to 6 hour time point and baseline to 24 hour timepoint were analyzed with a student's t-test to see if there was a significant difference between the two devices.

The results from this study met the pre-specified acceptance criteria for the primary endpoints: Change in Troponin I levels from baseline to 6 hours post shock and baseline to 24 hours post shock were not significantly different than LIFEPAK 12 (p=0.45 and p=0.89, respectively); and the mean histopathology injury score difference across all tissue sections was less than 0.4. There were also no significant differences in secondary endpoints including Creatine Kinases (CPK, CK-MB, and CK-MM), CBC and serum chemistry levels and ECG characteristics between the two groups.

Overall, this study provides the evidence that the ASSURE WCD waveform does not cause more injury than the LIFEPAK 12 waveform.

6 OVERALL STUDY PLAN

The objective of this study is to obtain human clinical experience with the ASSURE WCD defibrillation waveform in adult subjects. This is a multicenter single arm open label study using the ASSURE WCD defibrillation waveform to convert shockable rhythms (VT and VF). Subjects who consent to participate will be screened for eligibility before the enrollment visit is scheduled. The Investigator will verify eligibility at the time of enrollment.

Number of Subjects

This is a fixed study design with 20 subjects.

Study Sites

Subjects will be enrolled at up to 5 clinical sites in the United States where the procedures specified in Inclusion Criterion 3 are performed routinely. There is no minimum or maximum number of subjects that may be enrolled at each site.

Study Population

Patients who are scheduled for any of the procedures specified in Inclusion Criterion 3 (below).

Eligibility Criteria

Subjects who meet *all* Inclusion Criteria and *none* of the Exclusion Criteria may be enrolled in the study.

Inclusion Criteria:

1. Males or females, age ≥ 18 years
2. Able and willing to provide written informed consent before undergoing any study-related procedures
3. Scheduled for any of the following procedures:
    a. Electrophysiology study for induction of ventricular arrhythmias
    b. Non-invasive electrophysiology testing using an existing implantable defibrillator
    c. ICD replacement procedure during which induction of a ventricular arrhythmia is planned
    d. Ablation of ventricular tachycardia (patients undergoing ventricular tachycardia ablation in which ONLY a substrate modification approach is planned, with no intention of inducing a ventricular arrhythmia, should *not* be included)

Exclusion Criteria:

1. Any condition that by the judgement of the physician investigator precludes the subject's ability to comply with the study requirements
2. Pregnancy
3. Use of mechanical circulatory support (e.g. LVAD, Total Artificial Heart, intraaortic balloon pump or Impella)
4. Documented nonchronic cardiac thrombus
5. Atrial fibrillation or atrial flutter without adequate systemic anticoagulation
6. Critical aortic stenosis
7. Unstable coronary artery disease (CAD)
8. Recent stroke or transient ischemic attack (TIA)
9. Hemodynamic instability
10. Currently implanted Boston Scientific S-ICD (due to location of implant relative to test system)
11. Unstable Angina
12. New York Heart Association (NYHA) Class IV
13. Left Ventricular Ejection Fraction (LVEF) < 20%
14. Any medical condition that by the judgement of the physician investigator, patient participation in this study is not in the best interest of the patient
15. History of difficulty of ventricular arrhythmia induction
16. Amiodarone use within 3 months before the study procedure

Participation Period

Individual subject participation is during acute intra-procedural testing only. Subjects can be enrolled only once. Overall study duration will be approximately two months.

Subject Withdrawals

Subjects may withdraw from the study for any reason. Subjects may also be removed from the study due to adverse events or Investigator/Sponsor decision. Subjects who are withdrawn from the study will not be replaced.

Medical Monitor

The Medical Monitor (an independent physician not participating as a clinical investigator in the clinical study) will review and assess in conjunction with the Sponsor all AEs considered by the investigator to be at least possibly related to the use of the Test System to determine if they are reportable to the FDA as Unanticipated Adverse Device Effects (UADEs) per the definition in Section 10. The Medical Monitor will also review all Serious Adverse Events considered by the investigator to be at least possibly related to the use of the Test System to verify relatedness and the Serious classification.

Sponsor or Regulatory Agency Termination of the Study

Although the Sponsor intends to complete the study, the right is reserved to discontinue the study at any time for clinical or administrative reasons, or if required by the local regulatory authority.

End of Study

The end of study will be defined as the date of the last visit of the last subject unless open AEs remain in which case the end of study will be when that follow up is completed (the AE is resolved, or the subject is lost to follow up). A summary of the End of Study report will be sent to relevant regulatory authorities and Institutional Review Boards (IRBs) within 6 months of the end of the study.

7 STATISTICAL PLAN

Data Sources

- Case Report Form (CRF) Data will include: Subject ID, Subject Demographics, Confirmation of Eligibility, Cardiac Medical History, Procedure Data (induction attempt number, method, time, arrhythmia type, arrhythmia duration, arrhythmia rate, shock(s) delivered, shock converted), Adverse Events (AEs), Missing data rationale, Deviations and Investigator Signature page.
- EP Study/Testing, ICD changeout, or Ablation procedure documentation including ECG recordings in which conversion was attempted per Section 9 of this protocol, and procedure summary.

Study Objectives and Endpoints

Efficacy Objective: To evaluate conversion efficacy of the ASSURE WCD Waveform

Primary Endpoint: Estimated cumulative first and second shock VT/VF conversion efficacy ≥ 94%

Secondary Endpoint: First shock conversion efficacy (report only)

Safety Objective: To collect information on adverse events at least possibly related to use of the investigational Test System

Safety Endpoint: Summary of adverse events that are at least possibly related to use of the investigational Test System, including classification of Unanticipated Adverse Device Effects

Sample Size Considerations

The sample size set for this study has been scaled to obtain human clinical experience in a comparable number of subjects with attempted shock conversion as reported in the published US LifeVest registry study (Kutyifa V), and account for the potential for missing data (e.g., subjects who fail to have a ventricular arrhythmia induced). The sample size is not power-driven.

Analysis Populations

The primary endpoint will be analyzed using the data sets as defined below:

1. Intention to Treat (ITT) analysis population will include all subjects who signed informed consent and for whom the study procedure was initiated.
2. Per Protocol (PP) analysis population will include all subjects who met all eligibility criteria, signed informed consent, and for whom the procedure was completed as

specified in section 9 without deviation. The PP analysis population is a subset of the ITT population. If there are no differences in the ITT and PP analysis populations, separate analyses will not be presented.

The determination of any subjects excluded from the PP analysis population will be made prior to locking the study database for final analysis. All safety summaries will be based on the ITT analysis population.

Handling of Missing Data

CRFs will be monitored for accuracy and missing data, and queries will be issued to the sites for resolution. Every attempt will be made to maximize the likelihood of obtaining data to support the study endpoint

The table below lists anticipated situations which may occur resulting in missing data for the primary endpoint, and the steps that will be taken to minimize the occurrence.

Table 3: Steps to Minimize Missing Data for the Primary Endpoint

Source of missing data	Steps to minimize missing data
Unable to induce a ventricular arrhythmia	History of difficulty of ventricular arrhythmia induction has been specified in the exclusion criteria.
Unable to induce a ventricular arrhythmia with heart rate greater than 150 bpm	History of difficulty of ventricular arrhythmia induction has been specified in the exclusion criteria.
No induction attempted due to change in patient's hemodynamic status	Conditions which place the patient at perceived high risk have been specified in the exclusion criteria.
Rescue shock delivery before either a 1st or 2nd shock from the Test System	Protocol training will be provided for the investigator and EP staff.
Pacing or shock therapy from CIED delivered before either a 1st or 2nd shock from the Test System	1. Protocol training will be provided for the investigator and EP staff.<br>2. Instruction to program CIED therapies OFF is specified in the EP procedure (Section 9).
Pacing from external EP system delivered before either a 1st or 2nd shock from the Test System	Protocol training will be provided for the investigator, EP staff and operators
No shock delivered from the Test System due to procedure setup or operator error	1. Protocol training will be provided for the investigator, EP staff and operators.<br>2. Operators will be trained on the Test System.
No shock delivered from the Test System due to equipment failure	Each Test System must pass manufacturing test before shipment to the clinical site. In

	addition, the ASSURE WCD Monitor executes a self-test upon boot-up.

Occurrence of any of these situations shall be reviewed with the Sponsor and the Study PI prior to enrolling any additional subjects at that site. If there still are missing data despite efforts to minimize it the investigator will document on a CRF the reason/rationale for the missing data.

Primary Endpoint Analysis

The primary endpoint analysis will be performed after all subjects have completed the study and the study database has been cleaned, verified and locked. The data from all clinical sites that participate in this study will be combined so that the target sample size will be available for analysis.

The primary endpoint will be calculated as the ratio of the number of subjects with successful (first or second shock) arrhythmia conversion using the Test System to the number of total inductions attempted with shocks delivered by the Test System in the respective data set. A successful arrhythmia conversion is defined as termination of an induced ventricular rhythm (≥150 bpm) by first or second shock from the Test System to a non-shockable rhythm (rhythms other than VT or VF).

Performance criteria will be based on comparison to the published conversion efficacy point estimate of 94% reported for the ZOLL LifeVest (Wäßnig NK), which is the only comparable commercially-available device.

The Pass/Fail criteria will be based on the primary endpoint using the PP analysis population (See Table 3).

Table 4: Pass/Fail Criteria - Estimated Conversion Rate Based on Study Results

Successful Conversion	Estimated Conversion Rate	95% Confidence Interval
20 out of 20	100.0%	(83.2%, 100.0%)
19 out of 20	95.0%	(75.1%, 99.9%)
≤ 18 out of 20	Failure to Meet Primary Endpoint	N/A
19 out of 19	100.0%	(82.4%, 100.0%)
18 out of 19	94.7%	(74.0%, 99.9%)
≤ 17 out of 19	Failure to Meet Primary Endpoint	N/A
18 out of 18	100.0%	(81.5%, 100.0%)
17 out of 18	94.4%	(72.7%, 99.9%)
≤ 16 out of 18	Failure to Meet Primary Endpoint	N/A
17 out of 17	100.0%	(80.5%, 100.0%)
16 out of 17	94.1%	(71.3%, 99.9%)
≤ 15 out of 17	Failure to Meet Primary Endpoint	N/A

Two analyses of the primary endpoint will be conducted, one using the PP data set and a second using the ITT data set. The ITT analysis will include all 20 subjects regardless of missing data. If there is missing data, a tipping point analysis (including best- and worst-case scenarios) will be used to assess sensitivity of the analysis to the missing data. The difference between the conversion rate estimates from the PP and ITT analyses will be used to assess the magnitude of missing data bias.

Secondary Endpoint Analysis

This secondary endpoint is exploratory and does not have pre-specified performance criteria. Data will be summarized for both the ITT and PP analysis populations.

Analysis of Demographic and Baseline Data

Subject demographic and baseline data will be summarized for the PP analysis population. Continuous variables will be summarized using descriptive statistics (number of subjects, mean, standard deviation, median, minimum and maximum), and categorical variables will be summarized using the count and percentage of subjects in each category.

Safety Analysis

Safety will be analyzed based on the ITT analysis population. The assessment of safety will be based on the summaries of AEs, vital signs, physical examination findings, and ECGs. The Medical Monitor's assessment of seriousness and relatedness, rather than the investigator's assessment, will be used for summarizing and analyzing safety data.

Separate summaries of AEs related to use of the Test System and by severity will be prepared. The number of deaths and UADEs will also be presented, and events leading to discontinuation from the study will be listed and tabulated.

Continuous variables will be summarized by descriptive statistics, and categorical variables will be summarized using the count and percentage of subjects in each category.

Subject Disposition

Subject disposition including the number of subjects enrolled and number of subjects completing the study will be tabulated. The percentage of subjects completing the study will be based on the total number enrolled. Protocol deviations will also be summarized. Subject discontinuations and the reasons for discontinuation will also be summarized.

.

8 TEST SYSTEM

The Test System consists of a production-equivalent ASSURE WCD Monitor that will deliver the ASSURE defibrillation waveform (APPENDIX A). The ASSURE WCD Monitor will be from controlled inventory that is designated for clinical evaluations only. The Monitor will be connected to an ASSURE Defibrillation Adapter Module (ADAM) that allows use of a commercially-available Physio-Control LIFEPAK15 Therapy cable, and a commercially-available Physio-Control LIFEPAK15 5-wire ECG cable for patient connections.

Commercially available Physio-Control QUIK-COMBO RTS standard adhesive defibrillation electrodes will be applied in anterior and posterior positions that correspond to the defibrillation pad locations in the ASSURE WCD Garment. Standard adhesive defibrillation electrodes are used to limit interference with the electrophysiology procedure that the subject is otherwise undergoing. Otherwise, ASSURE WCD electrodes would also require the ASSURE Garment to hold the defibrillation electrodes in place on the body which may compromise the sterile field. Defibrillation efficacy using the QUIK-COMBO RTS electrodes is expected to be representative of defibrillation efficacy using ASSURE WCD electrodes since both types of electrodes have been designed and tested to meet the defibrillator electrode requirements as specified in IEC 60601-2-4 for electrode AC small signal impedance, AC large signal impedance and active area.

ECG signals will be monitored using 5 lead locations. Figure 1 shows an example of the Test System and EP monitoring setup to be used for the study, including the ASSURE WCD Monitor, the ADAM, and the EP monitor and backup rescue defibrillator. Figure 2 provides specific detail regarding the Test System defibrillation pad and ECG electrode placement.

Delivery of the ASSURE defibrillation waveform will be initiated by setting the ASSURE WCD Monitor to Manual Shock Mode. The Kestra Clinical Specialist will initiate the shock delivery on Investigator/Sub-Investigator command using the ASSURE Tablet that communicates with the ASSURE WCD Monitor via a Bluetooth connection (the Test System shock decision algorithm will not be used).

Figure 1: Example Test System and Monitoring Setup

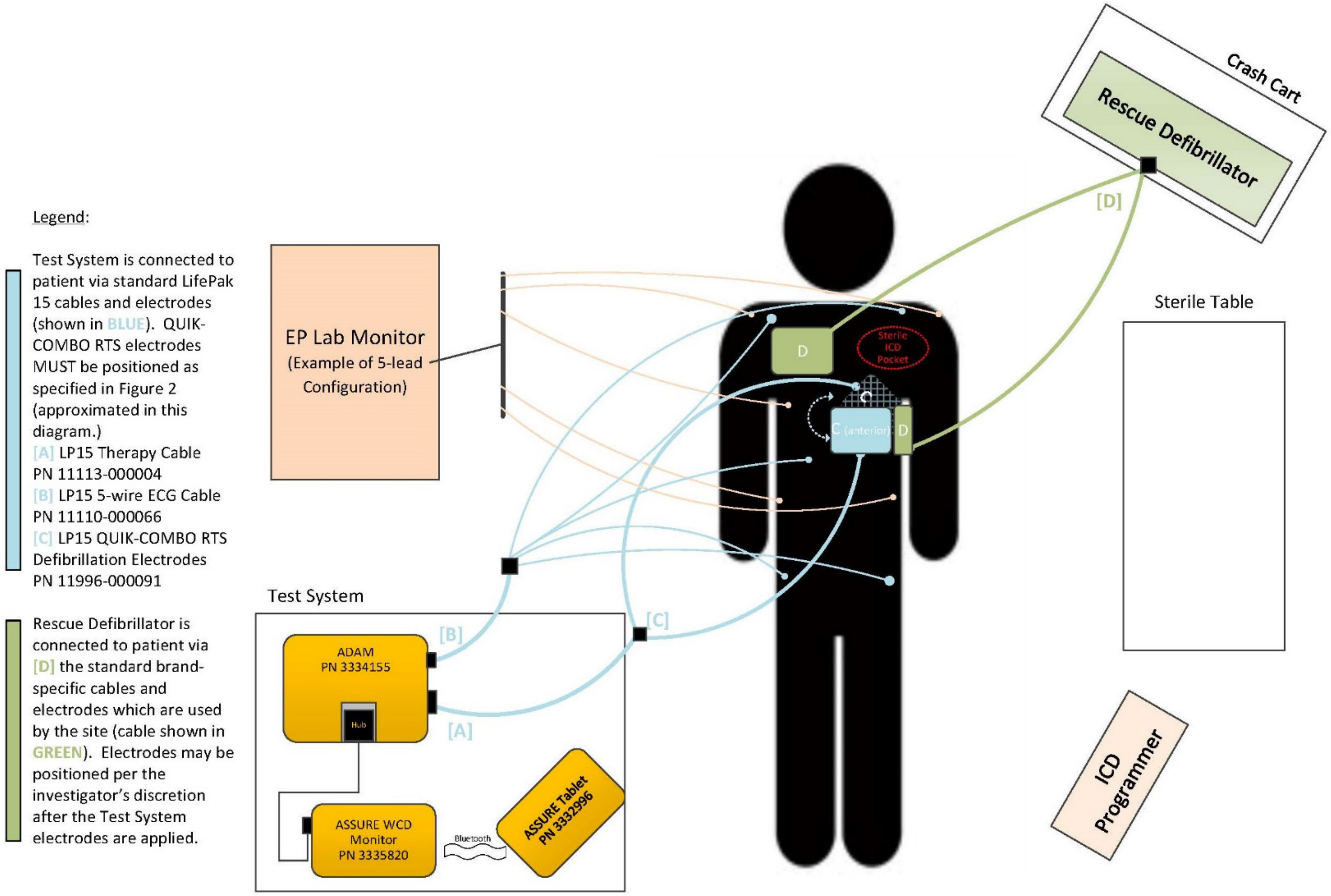

Figure 2: Test System Defibrillation Pad and ECG Electrode Placement

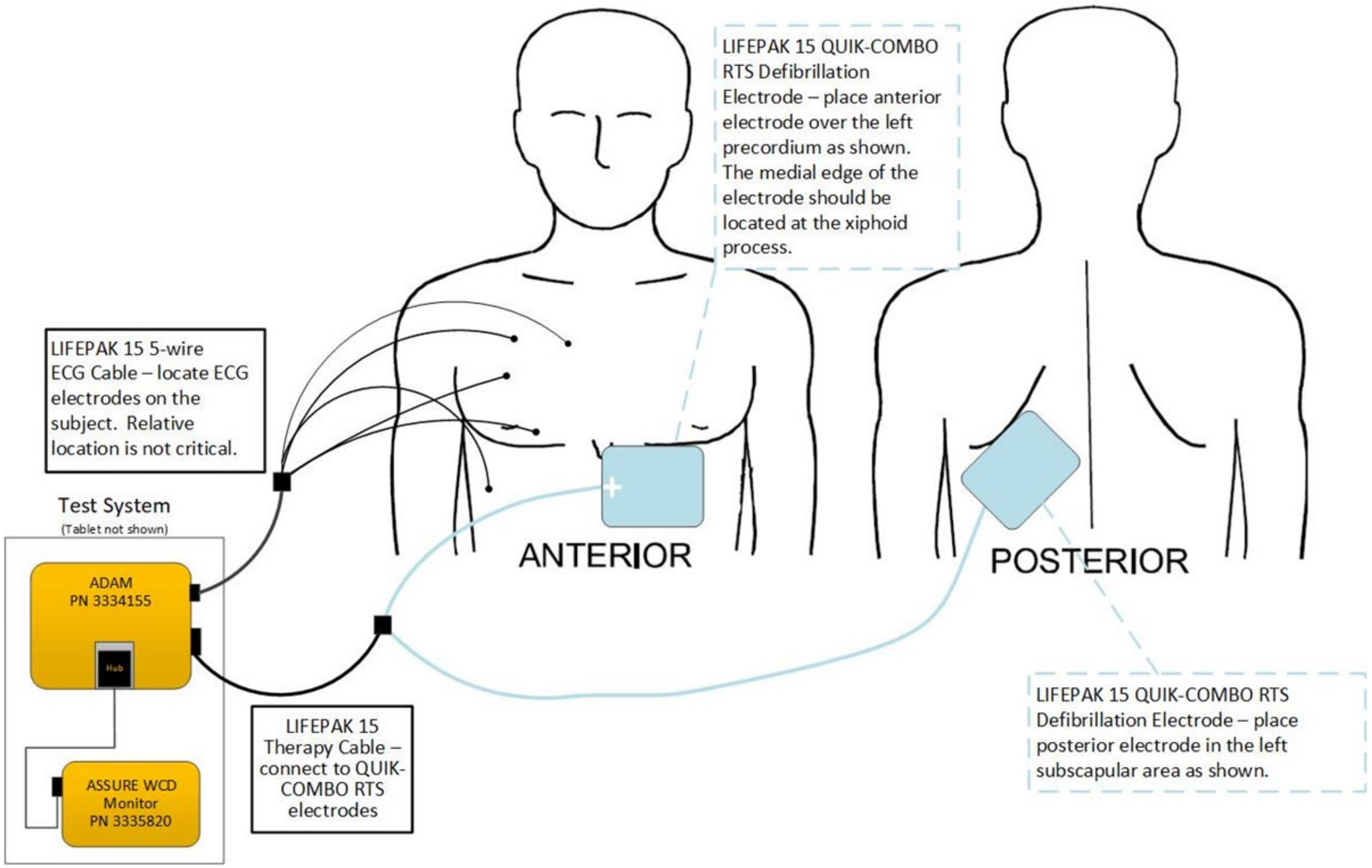

Safety and Device Accountability

Risks of this study have been identified through systematic hazard analysis, and the Sponsor has documented risk mitigation in a controlled Safety Risk Management Report.

Each ASSURE WCD Monitor and each ADAM will be assigned a unique serial number that is included on the labeling. Labeling also includes "For Investigational Use Only". The Sponsor will maintain tracking records for all system components.

All Test System components will be accounted for by Kestra Clinical staff.

Reuse of System Components

Adhesive ECG and defibrillation electrodes will be disposed of after use. Cables and Test System components will be cleaned between uses.

9 STUDY PROCEDURES

Informed Consent and Enrollment

Cardiac patients will be screened for eligibility by the clinical site staff members in advance of the procedure date. Eligible patients will be provided the informed consent form and allowed enough time to review the consent, and to have any questions they may have adequately addressed. Only patients who provide written informed consent to participate can be enrolled as study subjects. Subjects will be considered enrolled after they have provided Informed Consent and when the Test System is connected to the defibrillation electrodes in step 5 of the Electrophysiology procedure below.

Assessments

Demographic Data

Demographic data, including age, height, weight, gender, race, and ethnicity will be collected on the CRF.

Medical History

Medical history including information specified in the following list will be recorded on the CRF for each subject:

1. Disease process (ex., CAD, Dilated Cardiomyopathy, Hypertrophic Cardiomyopathy, Long QT/Brugada/CPVT)
2. Co-morbidities (ex., Diabetes, Renal disease, Hypertension, Hyperlipidemia, COPD)
3. Prior cardiac surgery (ex., PCI, CABG, other)
4. NYHA Class
5. The most recent left ventricular ejection fraction (%) measured by echocardiography, nuclear imaging (including MRI), or left ventricular angiography
6. History of Atrial arrhythmia
7. Prior Stroke/TIA
8. Baseline medications including any antiarrhythmic agents
9. Cardiac Implanted Electronic Device (i.e., pacemaker, ICD, CRT, ILR)
10. Shock therapy history from implantable or external devices

Clinical Laboratory Tests

Pregnancy testing will be performed by urine HCG for women of child bearing potential prior to enrollment.

Supply, Packaging, Labeling, and Storage

The Test System components will be provided by the Sponsor, including disposable defibrillation and ECG electrodes.

If Test System components are stored temporarily onsite, they must be stored at ambient room temperature in a securely locked cabinet or enclosure. Neither the Investigators nor any designees may provide devices to any individual not participating in this protocol.

Electrophysiology (EP) Procedure

The following procedure will be performed during a single study visit corresponding to the EP procedure. A Kestra Clinical Specialist must be present.

1. (Investigator or qualified designee) Explain the purpose and conduct of the study visits to the subject, answer the subject's questions, and obtain written informed consent. Provide the subject with a copy of the informed consent. Consent can be completed in advance of the procedure date, or on the day of the Study Visit.
2. (Investigator) Review inclusion/exclusion criteria prior to any procedural activities
3. (Investigator or qualified designee) Assign Subject ID number
4. (Investigator or qualified designee) Record demographic data and protocol-specified medical history on case report form
5. (Investigator or qualified designee in consult with Kestra Clinical Specialist) Position two sets of disposable adhesive defibrillation pads on the patient's torso:
    - Locate one set provided by the Sponsor in the left anterior and left-subscapular posterior positions to be connected to the Test System (see Figure 2). This configuration will be used to deliver the experimental therapeutic defibrillation shock(s).
    - Locate the other set of defibrillation pads (not provided by the Sponsor) for the backup rescue defibrillator) on the subject's torso according to physician preference but separated from the Test System defibrillation

pads to prevent arching or current shunt, and at least one inch away from a CIED if applicable.

- (Kestra Clinical Specialist) Obtain anterior and posterior photographs of the torso (not including subject's head/face) to document electrode placement.

6. (Kestra Clinical Specialist) Create a new patient in the ASSURE WCD Monitor, enter the subject ID number, and configure the ASSURE WCD Monitor to ***manual*** shock mode. (***Manual*** shock mode allows the Study Shocks to be delivered on command by the physician)
7. (Investigator or Sub-Investigator) Induce a single sustained episode of rapid VT or VF $\geq$ 150 bpm via the CIED or an electrophysiology catheter.
    - If this protocol is performed as part of an ICD replacement procedure, the test procedure must be performed prior to surgical opening of the CIED pocket.
    - If using a CIED for induction, ventricular therapy (i.e., ATP and Shocks) must be programmed OFF or temporarily suspended while therapy from the Test System is delivered.
    - If the arrhythmia is less than 150 bpm, convert the patient's arrhythmia using the backup rescue defibrillator and/or standard of care procedures.
    - Repeated induction attempts may be performed to induce the ventricular rhythm. The investigator may stop induction attempts at any point if in their judgement it is no longer in the best interest of the patient.
    - Conversion is defined as the presence of a non-shockable rhythm (rhythms other than VT or VF) after shock delivery
8. (Kestra Clinical Specialist under the direction of the Investigator or Sub-Investigator) If the Arrhythmia is estimated to be greater than 150 bpm, deliver a 170J shock from the Test System.
9. (Kestra Clinical Specialist under the direction of the Investigator or Sub-Investigator) Deliver a second shock at 170J from the Test System if the first shock is unsuccessful.
10. (Investigator or Sub-Investigator) If both attempts to convert using the Test System are unsuccessful, convert the patient's arrhythmia using the backup rescue defibrillator, implantable defibrillator and/or standard of care procedures.

Time to the first shock from the Test System will be approximately 6 seconds. Total time to an external rescue shock, if needed, is estimated to be approximately 20 seconds.

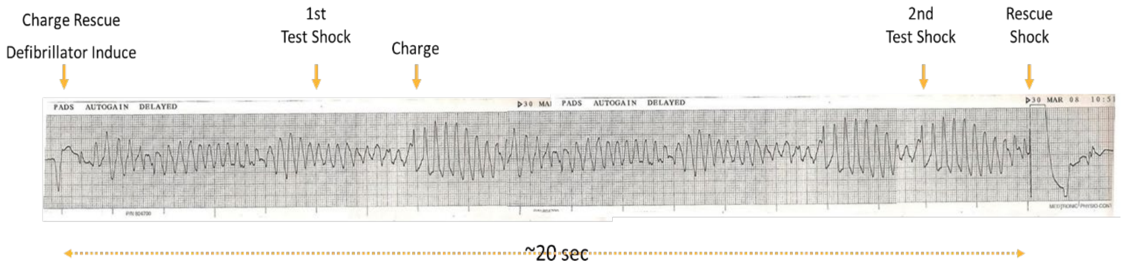

Figure 3: Time to shock

11. (Kestra Clinical Specialist) After conversion of the arrhythmia, disconnect the Test System from the adhesive pads.
12. Obtain copies of procedure documentation
    - Download the manual shock log from the tablet
    - Electronic or paper record of the ECG signals from the EP monitoring system (from induction through conversion)
    - Study-specific physician procedure report (de-identified)

The following situations are examples of protocol deviations:

a) Rescue shock delivered before a first or second Test System shock delivered
b) Therapy delivered from external EP system (e.g. ATP/pacing), or CIED (ATP/shock) before a first or second Test System shock delivered
c) No shock delivered from Test System (equipment failure or operator error)
d) No induction attempted after enrollment due to change in patient's hemodynamic status

Screen Failures

Subjects will be screen-failed if they do not meet eligibility, refuse consent to participate, or choose to withdraw consent to participate before they are enrolled as defined in paragraph 1 of this Section.

Handling of Withdrawals

When a subject is withdrawn from the study for any reason, the reason(s) for withdrawal will be recorded on the appropriate case report form (CRF). For any subject who is

withdrawn due to adverse events (AEs), the reason for withdrawal must be recorded as an AE and not any other reason.

In the event of a subject death during the study, the date of death (as listed on the death certificate) will be used as the date of study withdrawal.

It is vital to obtain follow-up data on any subject who is withdrawn because of an AE. In any case, every effort must be made to undertake protocol-specified safety follow-up procedures and the site principal investigator will be required to document that follow up.

10 ADVERSE EVENT REPORTING

Adverse events (AEs) as defined below will be monitored during the study procedure. AEs may be reported by the subject, discovered by Investigator questioning, or detected through physical examination or other means at the study visit. All AEs must be recorded on the CRF. The following information about each AE will be collected: description, severity, onset and resolution dates, seriousness, relationship to the Test System, action taken, and outcome.

Definitions

For the purposes of this study, an **AE** is defined as any untoward medical occurrence in a subject during the study that in the opinion of the investigator is at least possibly-related to use of the Test System during the EP procedures specified in Section 9.

A serious adverse event (**SAE**) is defined as any adverse event that:

- Leads to death
- Leads to serious deterioration in the health of a subject that:
    - Results in a life-threatening illness or injury, or
    - Results in a permanent impairment of a body structure or a body function, or
    - Requires in inpatient or prolonged hospitalization ≥ 24 hours, or
    - Results in medical or surgical intervention to prevent life-threatening illness or injury or permanent impairment to a body structure or function, or
- Leads to fetal distress, fetal death or a congenital anomaly or birth defect

Adverse Device Effect (ADE) is an adverse event related to the use of an investigational medical device (the Test System).

Anticipated Adverse Device Effects (AADEs) are those events related to the use of the Test System that are reasonably expected to occur as a result of the subject's participation in the study. The following are AADEs based on complications reported associated with defibrillation threshold testing (Wilkoff BL), complications associated with ICD pulse generator replacement (Poole JE), and the Test System engineering hazard analysis:

- Acute kidney injury
- Acute liver injury
- Blisters
- Burn
- Cardiac arrest
- Cardiac Perforation
- Cellulitis
- Central nervous system injury
- Death
- Deep vein thrombosis
- Depression of contractile function leading to worsening of heart failure
- Heart failure needing intravenous treatment with inotropes or diuretics
- Hematoma at catheter site requiring evacuation or long-term treatment with antiplatelet or anticoagulation medication
- Hemodynamic instability
- Hemothorax
- Hospital readmission
- Infection
- Itching under Test System defibrillation or ECG electrodes
- Lead dislodgment or malfunction (requiring re-operation)
- Myocardial Injury
- Pain persistent > 7 days
- Peripheral arterial embolus
- Persistent hypotension
- Pleural effusion
- Pneumothorax
- Prolonged hospitalization
- Pulseless electrical activity (PEA)
- Recurrent VT
- Refractory VF
- Respiratory arrest/depression
- Skin Rash
- Stroke or transient ischemic attack (TIA)
- Tamponade
- Thromboembolic events

An **Unanticipated Adverse Device Effect (UADE)** is any serious adverse effect on health or safety or any life-threatening problem or death caused by, or associated with the study device (the Test System), if that effect, problem, or death was not previously identified in nature, severity, or degree of incidence in this protocol; or any other unanticipated serious problem associated with the device that relates to the rights, safety, or welfare of subjects.

All AEs will be assessed by the investigator And the Medical Monitor according to:

- Whether the event is serious (SAE);
- The severity of the event (mild, moderate, severe):

  - Mild: Awareness of signs and symptoms, but easily tolerated; are of minor irritant type, causing no loss of time from normal activities; symptoms would not require medication or a medical evaluation; signs and symptoms are transient.
  - Moderate: Discomfort severe enough to cause interference with usual activities; requiring treatment, but not extended hospitalization or intensive care for the subject.
  - Severe: Incapacitating with inability to do work or usual activities; signs and symptoms may be systemic in nature or require medical evaluation and/or treatment; requiring additional hospitalization or intensive care (prolonged hospitalization).
- The relationship of the event to use of the Test System:
  - Possibly Related: There is a reasonable possibility that the AE may have been primarily caused by device use. The AE has a reasonable temporal relationship to the use of the device and follows a known or expected response pattern to device, but alternative etiology is equally or more likely compared to the potential relationship to the use of the device.
  - Probably Related: There is a reasonable probability that the AE may have been primarily caused by device use. The AE has a reasonable temporal relationship to the use of the device and follows a known or expected response pattern to device. Note: this definition assumes no alternative etiology is equally or more likely compared to the potential relationship to the use of the device.
  - Definitely Related: The AE has a strong causal relationship to device use. The AE follows a strong temporal relationship to the use of the device, follows a known response pattern to the device, and cannot be reasonably explained by known characteristics of the subject’s clinical state, other therapies, or change in clinical status during the electrophysiologic procedure.

Whenever possible, the Investigator should group signs or symptoms that constitute a single diagnosis under a single AE term (e.g. redness and swelling can be combined under skin irritation).

Baseline conditions are not considered adverse events unless the condition worsens because of Test System use.

Adverse Event Reporting Procedures

SAEs deemed at least possibly related to the use of the Test System will reviewed by the investigator and will be recorded on the CRF.

Upon awareness of a SAE, the study site must enter the data into the CRF within 3 working days. If the eCRF is not accessible the Sponsor or Sponsor representative must be notified of any SAE via telephone (1-866-694-7476).

UADEs must be reported by the investigator to the approving IRB as soon as possible, but not later than 10 working days after the investigator first learns of the effect.

Reporting UADEs to Regulatory Agencies

The Sponsor is responsible for the ongoing safety evaluation of the device. The Sponsor should promptly notify all concerned Investigators/Institutions and the FDA of findings that could adversely affect the safety of subjects, impact the conduct of the study, or alter IRB approval to continue the study. The Sponsor will report UADEs to the Food and Drug Administration (FDA) and other IRBs within ten days of the Sponsor's learning of them.

Follow-up of Adverse Events

Resolution means the subject has returned to the baseline state of health or the Investigator does not expect any further improvement in the subject's condition or does not expect worsening of the AE.

AEs deemed at least possibly related to the use of the Test System persisting at the time of the subject's study exit will be followed by the investigator until the events are resolved, the subject is lost to follow-up or the adverse events are otherwise explained. If further evaluations are required, the investigator will ensure that relevant additional information is documented in the CRF.

11 DATA HANDLING AND QUALITY ASSURANCE

Subject Identification Numbers

Each subject will be assigned a unique study identification (ID) number that will be a combination of the site ID and the subject ID when enrolled in the study. The first two characters of the ID number will reflect the study site number (e.g. S1). The remaining two digits will be separated by a hyphen from the site ID and will be assigned based on the enrollment sequence (e.g. the first subject enrolled at each site will be assigned 01). This number will be used on the Case Report Form (CRF) and on the ECG data collection file. The Sponsor will not collect Personal Health Information that would allow specific subject identification (e.g. date of birth, name, initials, medical record number) on the CRF or in the system data.

Case Report Forms

As part of the responsibilities assumed by participating in the study, the Investigator agrees to maintain adequate case histories for the subjects treated as part of the research under this protocol. The Investigator agrees to maintain an accurate CRF and source documentation as part of the case history for each subject. Source documentation may include chart notes, laboratory reports, and ECG strips.

All requested information is to be filled in on the CRF. If an item is not available or is not applicable, this should be indicated. Blank data fields should not be present unless otherwise directed.

CRFs must be reviewed, signed, and dated by the Investigator as required in a timely manner.

Study Data and Conduct Monitoring

The sponsor or sponsor representative will follow the study closely and will maintain necessary email, telephone, fax, and/or mail contact with the Investigators and study sites and will visit the study sites at periodic intervals. The study monitor will maintain current personal knowledge of the study through observation, review of study records and source documentation, and discussion of the conduct of the study with the Investigators and study site staff. During those visits, the study monitor will compare the subject data recorded in the CRF against source documents at the clinical site. The study monitor will specifically review CRFs to verify that the procedures were followed, e.g. was the test therapy delivered per protocol.

All aspects of the study will be carefully monitored by the Sponsor or its designee for compliance with applicable government regulations.

Inspection of Records

Investigators and institutions involved in the study will permit study-related monitoring, audits, IRB review, and regulatory inspection(s) by providing direct access to all study records. The Investigator or study site may be audited by the Sponsor or its representatives and/or regulatory agencies at any time. In the event of an audit, the Investigator agrees to allow the Sponsor, representatives of the Sponsor, the Food and Drug Administration (FDA), or other regulatory agency access to all study records.

The Sponsor will review case report form data and perform electronic edit checks on the data.

The Investigator should promptly notify the Sponsor of any audits scheduled by any regulatory authorities and promptly forward copies of any audit reports received to the Sponsor.

Study Record Retention

Government agency regulations and directives require that all study documentation pertaining to the conduct of a clinical study in the subject files as original source documents for the study must be retained by the Investigator until notified by the Sponsor in writing that retention is no longer necessary. Study documentation includes records of laboratory tests, clinical notes, and subject medical records. It is the responsibility of the Sponsor to inform the Investigator/institution as to when this documentation no longer needs to be retained.

Records containing subject medical information must be handled in accordance with the requirements of the applicable privacy rules and consistent with the terms of the subject authorization contained in the Informed Consent Form (ICF) for the study. Care should be taken to ensure that such records are not shared with any person or for any purpose not contemplated by the ICF. Furthermore, CRFs and other documents to be transferred to the Sponsor should be completed in strict accordance with the instructions provided by the Sponsor, including the instructions regarding the coding of subject identities. Should the Investigator wish to assign the study records to another party or move them to another location, written agreement must be obtained from the Sponsor. If requested, the Investigator will provide the Sponsor, applicable regulatory agencies, and applicable IRB with direct access to original source documents.

Investigators shall maintain all study-related documentation for a period of two (2) years following completion of the study, or the date of marketing approval or as per the local regulatory authority's guidelines and practices, whichever is longer. The Investigator should contact the Sponsor prior to destroying any study related documents.

Ethical Conduct of the Study

The Investigator agrees that the study will be conducted according to the applicable FDA regulations 21CFR 812.100 and 21CFR812.110. The Investigator will conduct all aspects of this study in accordance with all national, state, and local laws or regulations.

Informed Consent

Written informed consent in compliance with Title 21 of the CFR Part 50 shall be obtained from each subject prior to entering the study or performing any study procedures including pre-procedure preparation. An ICF template will be provided by the Sponsor or designee to investigative sites. The ICF will be submitted by the Investigator to his or her IRB for review and approval prior to the start of the study. If any institution-specific modifications to study-related procedures are proposed or made by the site, the ICF should be reviewed by the Sponsor and/or its designee, if appropriate, prior to IRB submission.

Before recruitment and enrollment, each prospective subject will be given a full explanation of the nature of the study and the action of the Test System. The subject will be informed that participation is voluntary and that they can withdraw from the study at any time. The subject will be allowed to read the approved ICF. Once the Investigator is assured that the subject agrees to participate in the study, the subject will be asked to give consent by signing the ICF.

The Investigator shall provide a copy of the signed and dated ICF to the subject. The original shall be maintained in the subject's medical records at the site.

Institutional Review Board

Federal regulations require that approval be obtained from an IRB prior to participation of subjects in research studies. Prior to subject enrollment, a signed copy of the IRB approval letter must be submitted to the sponsor. In addition, the protocol, informed consent form, advertisements to be used for subject recruitment, and any other written information regarding this study to be provided to the subject must be approved by the IRB. Documentation of all IRB approvals will be maintained by the site and will be available for review by the sponsor or its designee.

All IRB approvals should be signed by the IRB chairperson or designee and must identify the IRB by name and address, the clinical protocol by title and/or protocol number, and the date approval was granted.

The Investigator is responsible for submitting and obtaining initial and continuing review of the study at intervals not exceeding 1 year or as otherwise directed by the IRB. The investigator must provide written documentation of continued study review to the Sponsor or its designee.

12 ADMINISTRATIVE CONSIDERATIONS

Confidentiality

All laboratory specimens, evaluation forms, reports, and other records will be identified in a manner designed to maintain subject confidentiality. All records will be kept in a secure storage area with limited access.

Modification of the Protocol

The Investigator may implement a change from the protocol without prior Sponsor and IRB approval only to eliminate an immediate hazard to a subject, in which case, Sponsor and IRB must be notified of the change within 24 hours.

Amendments to the protocol must be submitted in writing to the IRB and approved prior to subjects being enrolled into an amended protocol.

Protocol Deviations

A protocol deviation occurs when the Investigator or subject has failed to adhere to significant protocol requirements. Specific categories to be documented include but are not limited to:

- Subjects who enter the study even though they do not satisfy the entry criteria
- Subjects who develop withdrawal criteria during the study but are not withdrawn
- Deviations from the Study Procedure specified in Section 9
- Any non-adherence to the protocol that results in a significant additional risk to the subject.

Any deviation involving eligibility criteria, or the Study Procedure specified in Section 9, shall be reviewed with the Study PI and the Sponsor prior to additional enrollment at that site. The Investigator must document and explain any protocol deviation in the subject's source documentation. Protocol deviations should be reported to the IRB according to their requirements. Protocol deviations may also be documented by the clinical monitor during monitoring visits and those observations will be reviewed with the Investigator.

The Investigator is responsible for enrolling subjects who have met protocol eligibility criteria. If the Investigator has a question concerning a subject who may not meet an entry criterion, they should contact the Sponsor to discuss the specifics. Waivers for protocol eligibility will not be granted in this study.

Study Reporting Requirements

By participating in this study, the Investigator agrees to submit reports of SAEs according to the time line and method outlined in the protocol. In addition, the Investigator agrees to submit periodic reports to his/her IRB as appropriate.

Financial Disclosure

Principal Investigators and Sub-Investigators are required to provide financial disclosure information to allow the Sponsor to submit the complete and accurate certification or disclosure statements required under Title 21 CFR 54. In addition, the Investigator must provide to the Sponsor a commitment to update this information promptly, if any relevant changes occur during the investigation and at the completion of the trial.

Financial Obligations

The Sponsor is not financially responsible for further testing/treatment of any medical condition that may be detected during the screening process. In addition, in the absence of specific arrangements, the Sponsor is not financially responsible for further treatment of the subject's disease.

Investigator Documentation

Prior to beginning the study, the Investigator will be asked to comply with 21CFR812 by providing the following essential documents, including but not limited to:

- An Investigator-signed Investigator Agreement page of the protocol
- An IRB -approved ICF, samples of site advertisements for recruitment for this study, and any other written information regarding this study that is to be provided to the subject
- IRB approval of the Investigator, the Protocol, and the subject-facing materials
- Curricula vitae for the Principal Investigator and each Sub investigator listed. Current licensure must be noted on the curricula vitae or a copy of the license provided. The curricula vitae must be signed and dated by the Principal Investigators and Sub investigators within 1 year of study start-up, indicating that they are accurate and current.
- Financial disclosure information to allow the Sponsor to submit complete and accurate certification or disclosure statements required under Title 21 CFR 54. In addition, the Investigators must provide to the Sponsor a commitment to promptly update this information if any relevant changes occur during the investigation, at the completion of the trial and 1 year following the completion of the study.

Clinical Trial Agreement

Payments by the Sponsor to Investigators and institutions conducting the study, requirements for Investigators' insurance, and other requirements are specified in the Clinical Trial Agreement.

Policy for Publication and Presentation of Data

Following completion of the study at all sites, data may be considered for reporting at a scientific meeting and/or for publication in a scientific journal. Draft manuscripts of any public disclosure shall be provided to the Sponsor 60 days prior to presentation or publication to enable the Sponsor to review and comment and take any steps necessary to protect its intellectual property rights, consistent with the Clinical Trial Agreement.

13 INVESTIGATOR AGREEMENT

I agree to conduct the study as outlined in the protocol entitled, "ASSURE WCD Clinical Evaluation – Conversion Efficacy Study (ACE-CONVERT)", and in accordance with generally accepted standards of Good Clinical Practice, and all applicable guidelines and government regulations including Title 21 CFR 54. I agree to provide the Sponsor with accurate financial information to allow the Sponsor to submit complete and accurate certification and disclosure statements as required by applicable regulations.

I have read and understand all sections of the protocol, including the section on ethical conduct of the study (Section 11) and administrative considerations (Section 12).

____

Principal Investigator's Name

____ ____________________

Principal Investigator's Signature Date

14 APPENDIX A - ASSURE DEFIBRILLATION WAVEFORM

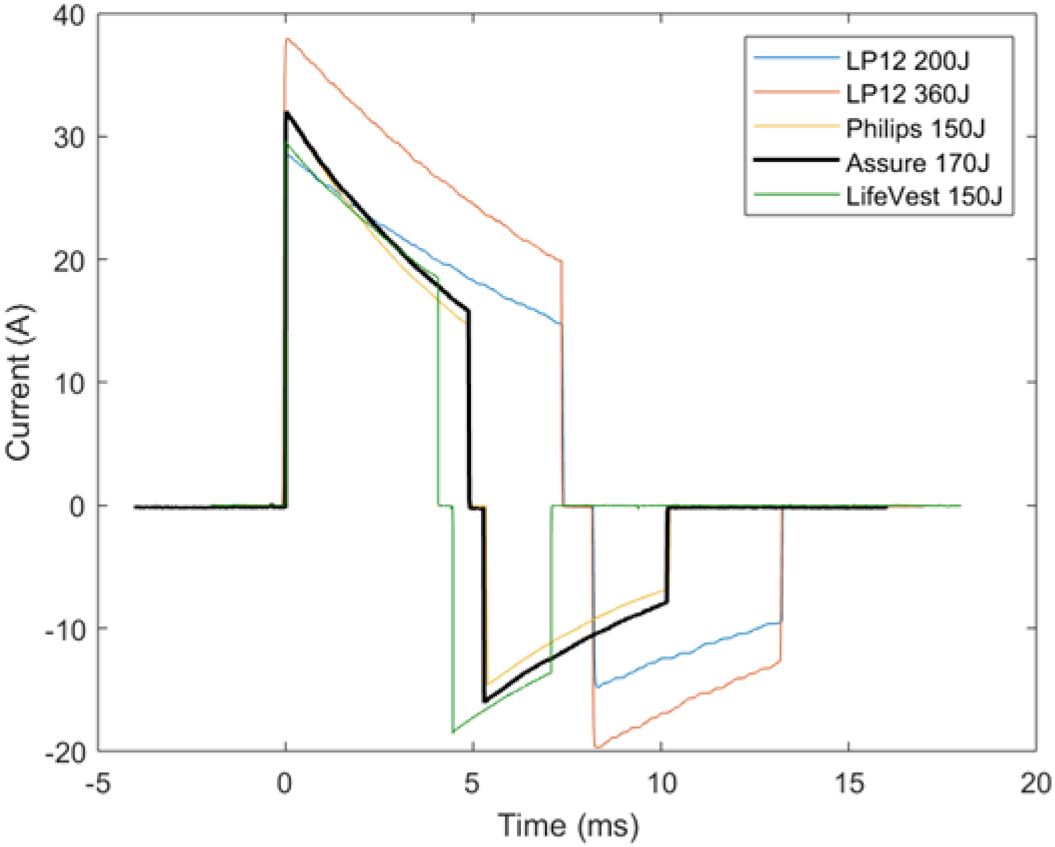

Figure 4: Defibrillation Waveform Profiles into 50 ohms

15 APPENDIX B - MEDICARE IDE STUDY CRITERIA

The study protocol describes the method and timing of release of results on all pre-specified outcomes, including release of negative outcomes and that the release should be hastened if the study is terminated early.

The study results on all pre-specified outcomes, including negative outcomes, will be submitted to ClinicalTrials.gov not later than one year after the study completion date, where the completion date is defined as the date that the final subject was examined or received an intervention for purposes of data collection for the pre-defined outcome measures. Results submission could be delayed if an extension is granted to the results submission deadline; however, the release of all results on pre-specified outcomes will be hastened if the study is terminated early. The final clinical study report of the conclusions of this study will be written within six (6) months of the closing of the database at the end of the study. Following completion of the study at all sites, data may be considered for reporting at a scientific meeting and/or for publication in a scientific journal.

The study protocol must describe how Medicare beneficiaries may be affected by the device under investigation, and how the study results are or are not expected to be generalizable to the Medicare beneficiary population. Generalizability to populations eligible for Medicare due to age, disability, or other eligibility status must be explicitly described.

Medicare patients that meet the study's eligibility requirements included will be enrolled in this study. It is not anticipated that the device under investigation will treat a Medicare population different than the demographics found in the investigators' general population for this same condition (*adult patients who are at risk of sudden cardiac arrest (SCA) and are not immediate candidates for or decline an implantable defibrillator*), including populations eligible for Medicare due to age (e.g., 65 years or older), disability, or other eligibility status. According to a science advisory prepared by the AHA and endorsed by the Heart Rhythm Society and European Society of Cardiology (ESC), Wearable Cardioverter Defibrillator (WCD) use is reasonable when there is an indication for an ICD but a temporary contraindication/interruption (eg, a waiting period, infection) to ICD care exists (class IIa), or when patients are awaiting more definitive treatment (eg, cardiac transplant candidate) (class IIa). VT/VF remains an important and potentially avoidable cause of SCD in high-risk patients. Moreover, WCDs are successful in terminating VT/VF in patients with an elevated risk of SCD and appear to be appropriate for use while long-term risk management strategies are being determined.[1] Additionally, it has been reported that the use of WCD use in patients ≥ 65 years old who are at risk of SCD is similar to that reported in general WCD patients; and, the majority of patients either recovered or proceeded to ICD implantation after ending use.[2] For these reasons, the results of this study are expected to be generalizable to the Medicare population primarily due to age (E.g., 65 years or older).

[1] Wearable Cardioverter-defibrillators for the Prevention of Sudden Cardiac Death: A Meta-analysis. *The Journal of Innovations in Cardiac Rhythm Management*, 9 (2018), 3151–3162

[2] The Wearable Cardioverter Defibrillator Protects Older Patients from Sudden Cardiac Death. Abstract 341. *Circulation*. 2014;130:A341

16 BIBLIOGRAPHY

Kutyifa V, Moss AJ, Klein H, et al. "Use of the Wearable Cardioverter Defibrillator in High-Risk Cardiac Patients: Data from the Prospective Registry of Patients Using the Wearable Cardioverter Defibrillator (WEARIT-II Registry)." *Circulation* 132 (2015): 1613-1619.

Poole JE, Gleva MJ, Mela T, et al. "Complication rates associated with pacemaker or implantable cardioverter-defibrillator generator replacements and upgrade procedures: results from the REPLACE registry." *Circulation* (2010): 122(16): 1553-61.

Wäßnig NK, et al. "Experience with the Wearable Cardioverter-Defibrillator in Patients at High Risk for Sudden Cardiac Death." *Circulation* (2016): 134:635-643.

Wilkoff B, Fauchier L, Stile M, et al. "2015 HRS/EHRA/APHRS/SOLAECE expert consensus statement on optimal implantable cardioverter-defibrillator programming and testing." *J Arrhythm* 32 (2016): 1-28.

Wilkoff BL, et al. "2015 HRS/EHRA/APHSR/SOLAECE expert consensus statement on optimal implantable cardioverter-defibrillator programming and testing." *Heart Rhythm* (2016): Vol 13, No 2, e71.
